# Diagnosis and functional prediction of microbial markers in tumor tissues of sporadic colorectal cancer patients associated with the MLH1 protein phenotype

**DOI:** 10.3389/fonc.2022.1116780

**Published:** 2023-01-23

**Authors:** Anchao Zhu, Yingying Liu, Zongmin Li, Ying He, Lijing Bai, Youtian Wu, Yuying Zhang, Ying Huang, Ping Jiang

**Affiliations:** ^1^ Department of Pathology, Harbin First Hospital, Harbin, China; ^2^ Department of Pathology, Harbin Medical University, Harbin, China; ^3^ Department of Pathology, Heilongjiang Provincial Hospital, Harbin, China; ^4^ Department of Gastroenterology, Harbin First Hospital, Harbin, China; ^5^ Department of Laboratory Diagnosis, First Affiliated Hospital of Harbin Medical University, Harbin, China

**Keywords:** colorectal cancer, 16S rRNA gene, biomarker, MLH1, microbiome

## Abstract

**Objective:**

Most patients with sporadic colorectal cancer (SCRC) develop microsatellite instability because of defects in mismatch repair (MMR). Moreover, the gut microbiome plays a vital role in the pathogenesis of SCRC. In this study, we assessed the microbial composition and diversity of SCRC tumors with varying MutL protein homolog 1 (MLH1) status, and the effects of functional genes related to bacterial markers and clinical diagnostic prediction.

**Methods:**

The tumor microbial diversity and composition were profiled using high-throughput sequencing of the 16S ribosomal RNA (rRNA) gene V4 region. Phylogenetic Investigation of Communities by Reconstruction of Unobserved States (PICRUSt2) software and BugBase tool were used to predict the functional roles of the microbiome. We aimed to construct a high-accuracy model to detect and evaluate the area under the receiver operating characteristic curve with candidate biomarkers.

**Results:**

The study included 23 patients with negative/defective MLH1 (DM group) and 22 patients with positive/intact MLH1 (IM group). Estimation of alpha diversity indices showed that the Shannon index (p = 0.049) was significantly higher in the DM group than in the controls, while the Simpson index (p = 0.025) was significantly lower. At the genus level, we observed a significant difference in beta diversity in the DM group versus the IM group. Moreover, the abundance of *Lachnoclostridium* spp. and *Coprococcus* spp. was significantly more enriched in the DM group than in the IM group (q < 0.01 vs. q < 0.001). When predicting metagenomes, there were 18 Kyoto Encyclopedia of Genes and Genomes pathways and one BugBase function difference in both groups (all q < 0.05). On the basis of the model of diagnostic prediction, we built a simplified optimal model through stepwise selection, consisting of the top two bacterial candidate markers (area under the curve = 0.93).

**Conclusion:**

In conclusion, the genera Lachnoclostridium and Coprococcus as key species may be crucial biomarkers for non-invasive diagnostic prediction of DM in patients with SCRC in the future.

## Introduction

According to Cancer Statistics, 2021, colorectal carcinoma (CRC) is the third most prevalent cancer in the United States, with sporadic and microsatellite-stable (MSS) CRC accounting for approximately 80–85% of the cases and microsatellite-instability (MSI) accounting for the rest (1, 2). In addition, sporadic CRC (SCRC, a subtype of CRC) development is related to several potential etiological factors, such as lifestyle, methylation, genetics (BRAF or MSI mutation), and the gut microbiome, which affect the prognosis and survival of patients with SCRC (3–5). However, the relationship between the mismatch repair (MMR) system and microbial markers, which is the main factor affecting the prognosis and survival of patients with SCRC, remains unclear. Accumulating evidence indicates that the gut microbiome and its metabolites are closely related to the development and progression of SCRC (6).

According to the National Comprehensive Cancer Network^®^ (NCCN) Clinical Practice Guidelines in Oncology (NCCN Guidelines), bevacizumab is suitable as the first-line treatment before maintenance therapy for patients with unresectable tumors such as metastatic CRC (7, 8). Retrospective studies have found that although patients with high-MSI (MSI-H) CRC generally have a better prognosis than those with low MSI (MSI-L) or MSS CRC, they do not benefit from postoperative adjuvant 5-fluorouracil based chemotherapy (9). Recently, cytotoxic T-lymphocyte-associated protein 4 (CTLA-4) and programmed death 1 (PD-1) blockers have attracted widespread attention as immunotherapy agents and have been proven to be effective treating several types of solid tumors (10). The gut microbiome has a prominent influence on the clinical effects of solid tumor treatment using immunotherapy (11, 12). However, patients with SCRC have not benefitted from immunotherapy, which is likely to be effective only if the tumor exhibits MSI (13). Therefore, an in-depth study of the composition and function of the gut microbiome may help determine the therapeutic direction for patients with SCRC.

Currently, defective MMR genes such as MLH1, MSH2, MSH6, and PMS2 can be detected in tumor and normal tissues (usually blood or adjacent normal tissues) using immunohistochemistry (IHC) and polymerase chain reaction (PCR) (14). However, researchers have discovered that PCR-based MSI detection methods are subpar (15); hence, the IHC method is preferred in clinical pathology practice. According to mutation patterns, deficient mismatch repair (DMMR) proteins are generally considered equivalent to MSI-H, and proficient mismatch repair (PMMR) proteins are considered equivalent to MSI-L or MSS. Therefore, MSI is a molecular manifestation of defects in the MMR system and acquired abnormal methylation of the MLH1 gene promoter region is considered the main cause of SCRC (16). MSI-H/DMMR is a tumor marker in patients with Lynch syndrome; however, data from examining the MMR gene somatic defect revealed a sporadic event in 70–85% (14) of cases. With increasing evidence of a subtle link between the phenotypes of specific proteins in patients with SCRC and the gut microbiome, differences in gut microbiome diversity between MMR-deficient and MMR-nondeficient SCRC cohorts need further exploration.

The number of gut microorganisms in humans is over 100 trillion, and some of them participate in the host’s metabolic activities and immune regulatory functions (17). Research has shown that intestinal bacteria play a critical role in determining intestinal health and that specific microbes, such as *Fusobacterium* (*F.*) *nucleatum* (18, 19) and *Peptostreptococcus anaerobius* (20), promote colon tumor development. Using 16S rRNA gene sequencing, several microbial species have been found to pose a carcinogenic risk to host intestinal epithelial cells. Previously, *Escherichia coli NC101* was shown to regulate metabolic pathways and activate oncogenes, which can cause inflammatory changes in intestinal tissue (21). Furthermore, *F. nucleatum* was shown to influence host immune responses according to the MSI status (22, 23). Thus, microbial infection, and chronic inflammation are potential risk factors for intestinal malignancy, but the interactions of related molecular phenotypes (such as DNA repair proteins) with the gut microbiome remain unclear.

This study explored the diversity and composition of the gut microbiome from different perspectives and determined the possible functional pathways to gather relevant data for subsequent microbiome research related to MLH1 deficiency. PCR and IHC are used to methods evaluate tissues obtained after surgical resection, requiring patients to undergo invasive procedures. Therefore, we explored the feasibility of developing a non-invasive prediction method with high predictive value and designed an experimental study to identify bacterial gene markers in the tumor tissue that are strongly associated with MLH1 protein deficiency.

## Materials and methods

### Human specimens and procedure

**Figure 1:**
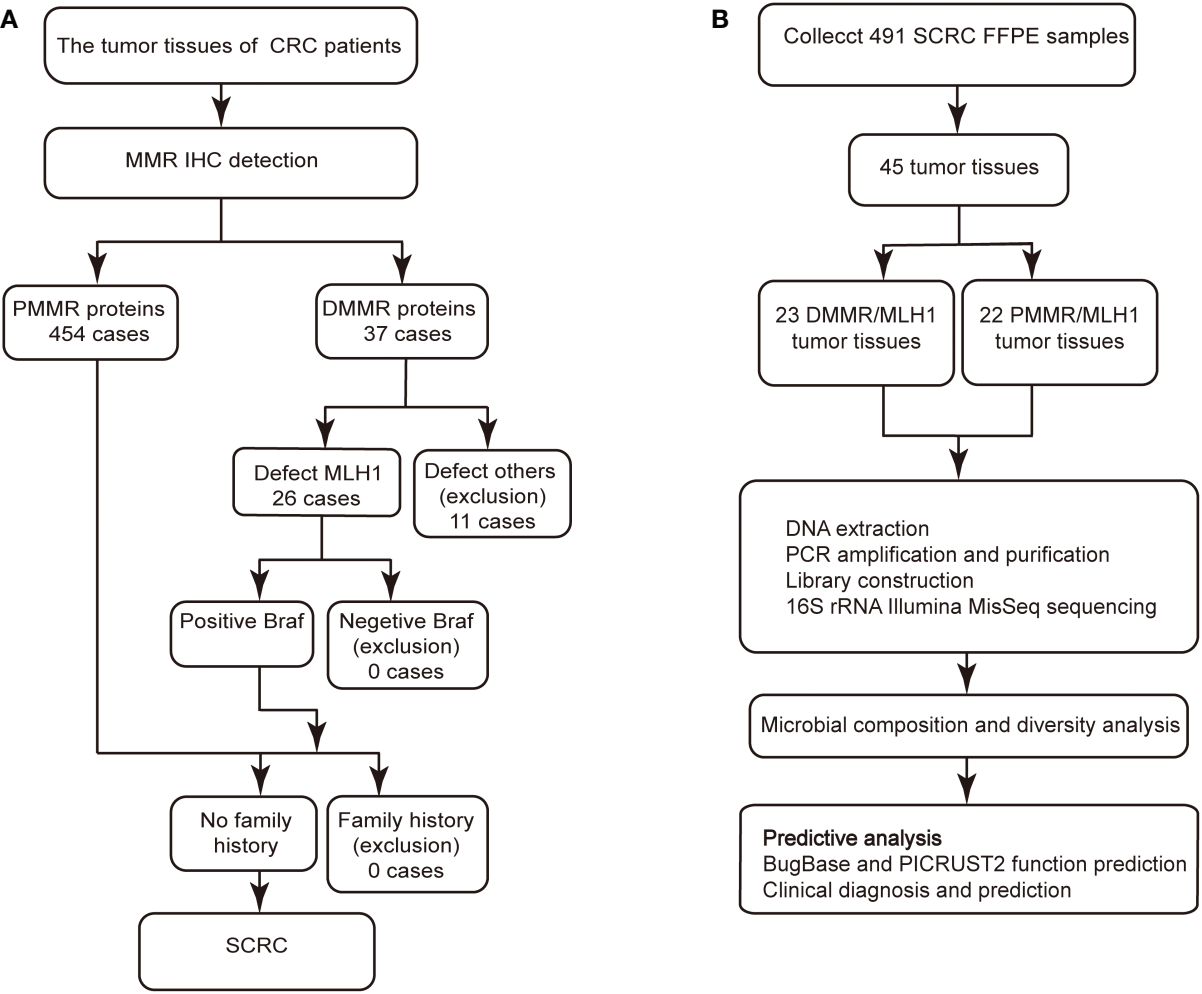
IHC staining and Gram staining were performed between the two groups. **(A)** Screening procedure for SCRC patients by MMR immunohistochemistry. **(B)** Study design and flow diagram. SCRC, sporadic colorectal carcinoma; MMR, mismatch repair; DMMR, deficient mismatch repair; PMMR, proficient mismatch repair.

In this study, BRAF mutations were an important aspect of screening for patients with MSI-positive SCRC after CRC MLH1 deletion but not for patients with MSI-negative (24–27). Subsequently, the data on family history were combined with the screening data to determine whether these patients had SCRC. Accordingly, patients with SCRC were screened on the basis of the Chinese expert consensus on clinical diagnosis, treatment, and pedigree management of hereditary CRC as recommended by the Colorectal Cancer Professional Committee of the Chinese Anti-Cancer Association (28) (**Figure 1A**). Following the recommended procedure, we designed the scheme for this study (**Figure 1B**). From 2020 to 2021, we retrospectively evaluated the pathology data of 491 patients from Northeast China, including 112 and 379 patients from the Harbin First Hospital and from the Heilongjiang Provincial Hospital, respectively. Basic clinical demographics and clinicopathological data for all the patients were obtained from the hospital’s electronic medical records. Formalin-fixed paraffin-embedded (FFPE) tissue specimens were obtained by surgical resection in untreated patients with SCRC. The inclusion criteria were as follows: no history of drug consumption such as antibiotics or probiotics within the past 3 months, no family history of Lynch syndrome, no surgical history, or no treatment history. This study was approved by the Ethics Committee of Harbin First Hospital (Approval No. Q2021-064).

### IHC and gram staining

For IHC staining, an SP (Rabbit) IHC Kit (Gene Tech, Shanghai, China) was used, according to the manufacturer’s instructions. Staining was performed as previously described (29). and all surgically resected tumor tissues were immediately fixed in 10% neutral formaldehyde solution. For the IHC procedure, 4-µm sections were used. MLH1, PMS2, MSH2, MSH6, and BRAF monoclonal antibodies were added to each sample, and the sections were fixed with the neutral resin for observation. Xylene was used in the deparaffinizing process of the slides, followed by graded ethanol for hydrated. Gram staining (Baso, Zhuhai, China) was performed on all tissue sections of equal thickness by referring to the reagent preparation instructions. The stained tissue slides were observed using the LEICA DM3000 light microscope at 400× and 1000× magnifications. The diagnostic results of all clinicopathological sections were confirmed through double-blind observation by three pathologists with deputy senior or higher titles. External positive control tissue was included on each slide to ensure the reliability of the experimental results.

### DNA extraction and 16s rRNA gene sequencing

Bacterial genomic DNA was extracted from each sample using the FastDNA^®^ SPIN Kit for Soil (MP Biomedicals, United States). Ten 8-µm FFPE tissue sections were dewaxed, bacterial genomic DNA was extracted, and the extracted DNA was purified in a 1.5 mL Eppendorf tube using 100% ethanol precipitation. DNA quantification and purity was performed using the NanoDrop 2000 UV-vis spectrophotometer (Thermo Scientific, Wilmington, USA). The integrity and size of the DNA fragments were determined using 1% agarose gel electrophoresis. All 16S rRNA genes were extracted using previously developed methods (30–32) and were stored at −20°C until further processing.

PCR was used to amplify the 16S (variable region 4 [v4]) rRNA genes using the 515F–806R primer, which is a general primer (forward: 5′-GTGCCAGCMGCCGCGG-3′; reverse: 5′-GGACTACHVGGGTWTCTAAT-3′). The PCR products were extracted from 2% agarose gel electrophoresis and purified using the AxyPrep DNA Gel Extraction Kit (Axygen Biosciences, Union City, CA, USA). Subsequently, the amplicons were quantified using Quantus™ Fluorometer (Promega, USA). We used the TruSeq Nano DNA LT Library Prep Kit to prepare DNA library. In accordance with the manufacturer’s instructions, the prepared library was checked using the 2x300 bp paired-end protocol (Majorbio Bio-Pharm Technology, Shanghai, China) for subsequent raw sequencing data on the Illumina MiSeqPE300 platform (Illumina, San Diego, USA).

The raw data were demultiplexed and quality-filtered using the FASTQ format (33) and were merged using FLASH (version 1.2.11^1^) (34) according to the minimum identity of the sample sequence within a cluster. Refer to Yu et al. (35), the data processing needed to proceeded the corresponding criteria. The methods of USEARCH (version 11.0^2^) were used for the operational taxonomic unit (OTU) clustering analysis with a 97% threshold. The ribosomal database project classifier (36) (version 2.13^3^) was used to against the SILVA (version 138^4^)/16S rRNA database. The OTU representative sequences were annotated with a confidence threshold of 70% (37), indicating that the similarity of more than 70% is the same species.

### Prediction of functional modules

The annotated OTU matrix of microbial characteristics was predicted using the BugBase^5^ microbiome analysis tool based on nine phenotypic categories. Moreover, by integrating the standard functional gene content of species using the PICRUSt2 software (version 2.2.0^6^), the proportional abundance spectrum of the Kyoto Encyclopedia of Genes and Genomes (KEGG) database in SCRC samples was determined. The Wilcoxon signed-rank test was used as the inspection method.

### Clinical diagnosis prediction analysis

Random forest (RF) analysis was used to classify and predict the accuracy and adaptability of the data, and the receiver operating characteristic (ROC) validation algorithm was applied. The software used in this model prediction analysis included the random forest package (R) and plotROC package (R). Based on previous descriptions (38), the number of decision trees was set to 500, and the relative abundance of OTUs was used for data standardization processing. The method of 10-fold cross-validation was simultaneously used to evaluate the important ranking variables to determine the minimum predicted number of taxa. The ROC curve was used to predict clinical diagnostic biomarkers in the microbiome of patients with CRC, and confidence intervals (CIs) were set at 95%for the CRC risk. The area under the ROC curve was interpreted using the following criteria: the area under the curve (AUC) had a low accuracy when it was between 0.5 and 0.7, a certain accuracy between 0.7 and 0.9, and a high accuracy above 0.9. An AUC of 0.5 indicated that the diagnostic method was completely ineffective and had no diagnostic value.

### Statistical analysis

Categorical variables in quantitative data were compared using the chi-square and one-way analysis of variance tests, with the SPSS software (version 21.0). Statistical significance was set at p < 0.05. The dilution curve was plotted using the R software, with the number of OTUs corresponding to each sample. Alpha diversity analysis was used to determine the diversity and richness of the microbial colonies using the Mothur (version 1.30.2) software package. Principal coordinate analysis (PCOA) was used to evaluate the structural distribution of microbial colony samples through dimensionality reduction so as to simplify the structure of sequencing data on the matrix of Bray Curtis and weighted UniFrac distances. The heatmap was generated according to the abundance of genera using R. In this study, CIs were calculated using a two-tailed test. To assess the differences in bacterial abundance of various groups, we performed the Mann−Whitney U test for different microbial system classification units, and the adjusted p value (q value) < 0.01, which was obtained through the false discovery rate (FDR) step-up procedure, was considered significant for multiple comparisons.

## Results

### MMR mutation and BRAF mutation analysis with CRC tumor tissues

**Figure 2:**
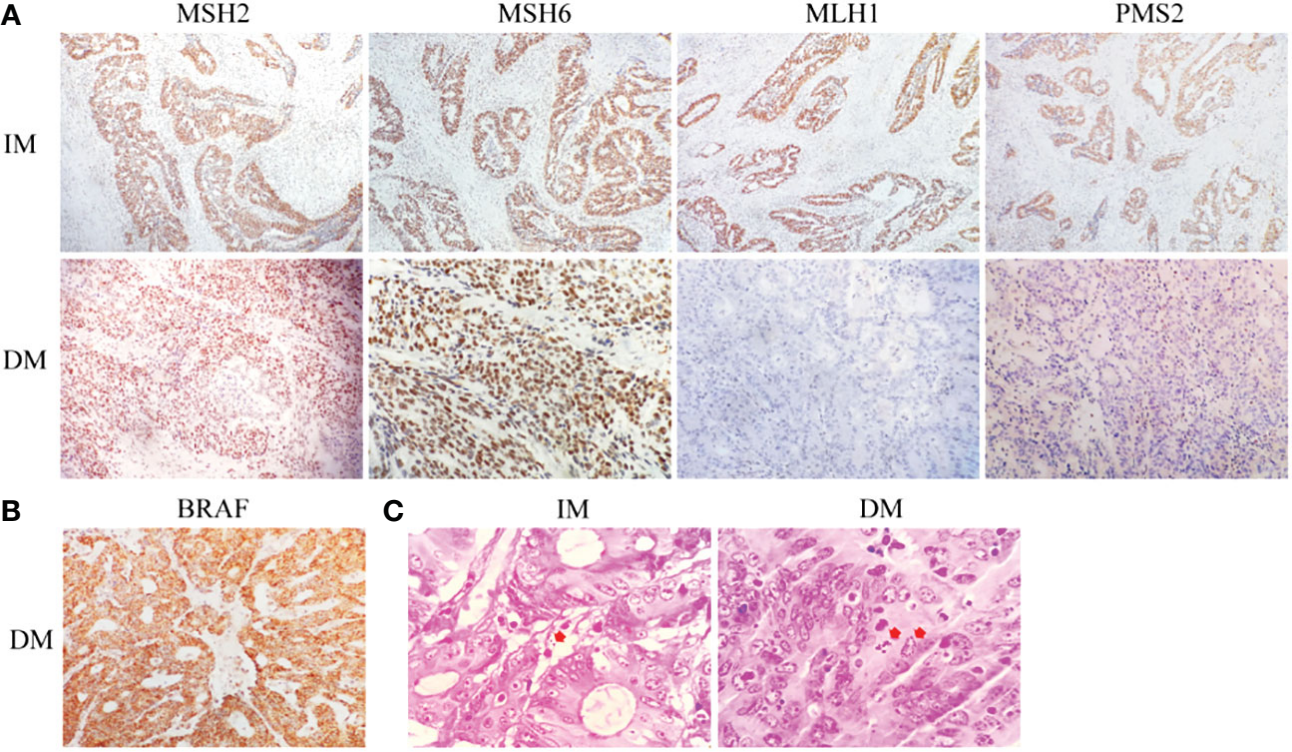
Histological staining of tumor specimens in both groups. **(A)** The corresponding IHC staining, followed by MSH2, MSH6, MLH1, and PMS2. **(B)** BRAF staining. **(C)** Gram staining (the arrow indicates piles of bacterial infections).

We conducted IHC tests on all surgically resected specimens obtained from adult patients with CRC in which the positive or negative expression of MMR proteins (MLH1, PMS2, MSH2, or MSH6) was observed in the cell nuclei. The positive expression of MMR proteins showed staining of tumor nuclei and a brown color, whereas the negative expression showed no staining (**Figure 2A**). The loss of heterodimeric binding of MLH1 with PMS2 (or of MSH2 with MSH6) indicates an underlying mutation in PMMR, dominated by MLH1 (or MSH2). Of the 491 SCRC cases, 454 (92.5%) and 37 (7.5%) involved PMMR and DMMR proteins, respectively. In DMMR proteins, MLH1 deficiency (simultaneous deletion of PMS2) was the most prevalent mutation and was identified in 70.3% (26/37) of the SCRC cases, whereas only 7 cases (18.9%) showed PMS2 deficiency (**Table 1**). Of these, the expression patterns of 3 cases with MLH1 deficiency were not representative, leaving 23 cases for further analysis. Meanwhile, the proportion of protein deletions in MSH2 and MSH6 was lower, accounting for only 10.8% (4/37) of the cases. Subsequently, we performed a supplementary study of BRAF markers (unpublished data), showing that all defective MLH1 cases had BRAF-positive expression (**Figure 2B**); that is, the presence of BRAF mutations can be considered as an inclusion criterion for patients with SCRC.

**Table 1:** Amount of the patterns in 37 SCRC patients with DMMR.

DMMR proteins	SCRC (No. of patients)	Percentage (%)
MLH1(-)	26	70.3
PMS2(-)	7	18.9
MSH2(-)	3	8.1
MSH6(-)	1	2.7
Total	37	100

DMMR, deficient mismatch repair; SCRC, sporadic colorectal cancer.

### Demographic characteristics and screening of SCRC patients with different MLH1 levels

The role of tumor microbiome composition in the clinical prognosis of SCRC tumor tissues that manifest with varying MLH1 status was explored. This study included 23 DMMR/MLH1 patients and 22 stage-matched PMMR/MLH1 controls. Additionally, patients in both groups were matched with the provided general information (age and sex) and clinicopathologic characteristics (including tumor diameter, differentiation, TNM stage, and American Joint Committee on Cancer stage), as shown in **Table 2**. There were no significant differences in these variables between the two groups (all p > 0.05), except for tumor size (p < 0.01). Gram staining showed the morphology and structure of bacteria in the surrounding and adjacent stroma of cancer cells (**Figure 2C**). Subsequently, bacterial DNA was extracted from these specimens, and taxonomic profiling *via* 16S rRNA gene sequencing was performed.

**Table 2:** Clinicopathological features of SCRC patients.

Characteristics	Total (n=45)	DM (n=23)	IM (n=22)	p-value
Age(years)	65.00 ± 11.04	64.30 ± 13.10	65.73 ± 8.97	0.674
Gender				0.884
Male	23	12	11	
Female	22	11	11	
Tumor Diameter(cm)	4.9 ± 1.6	5.5 ± 1.8	4.2 ± 1.0	0.005^**^
Differentiation				0.936
Well to Moderate	35	18	17	
Poor	10	5	5	
Primary Tumor				0.699
Low T-stage(T1/T2)	7	3	4	
High T-stage (T3)	38	20	18	
Lymph Node Metastasis				0.314
Node Negative(N0)	34	19	15	
Node Positive(N1/N2)	11	4	7	
Distant Metastasis				0.233
M0	43	23	20	
M1	2	0	2	
AJCC Stage				0.314
I–II	34	19	15	
III–IV	11	4	7	

DM, defective MLH1; IM, intact MLH1. ^**^p < 0.01.

### Comparison of microbial diversity between defective and intact MLH1

**Figure 3:**
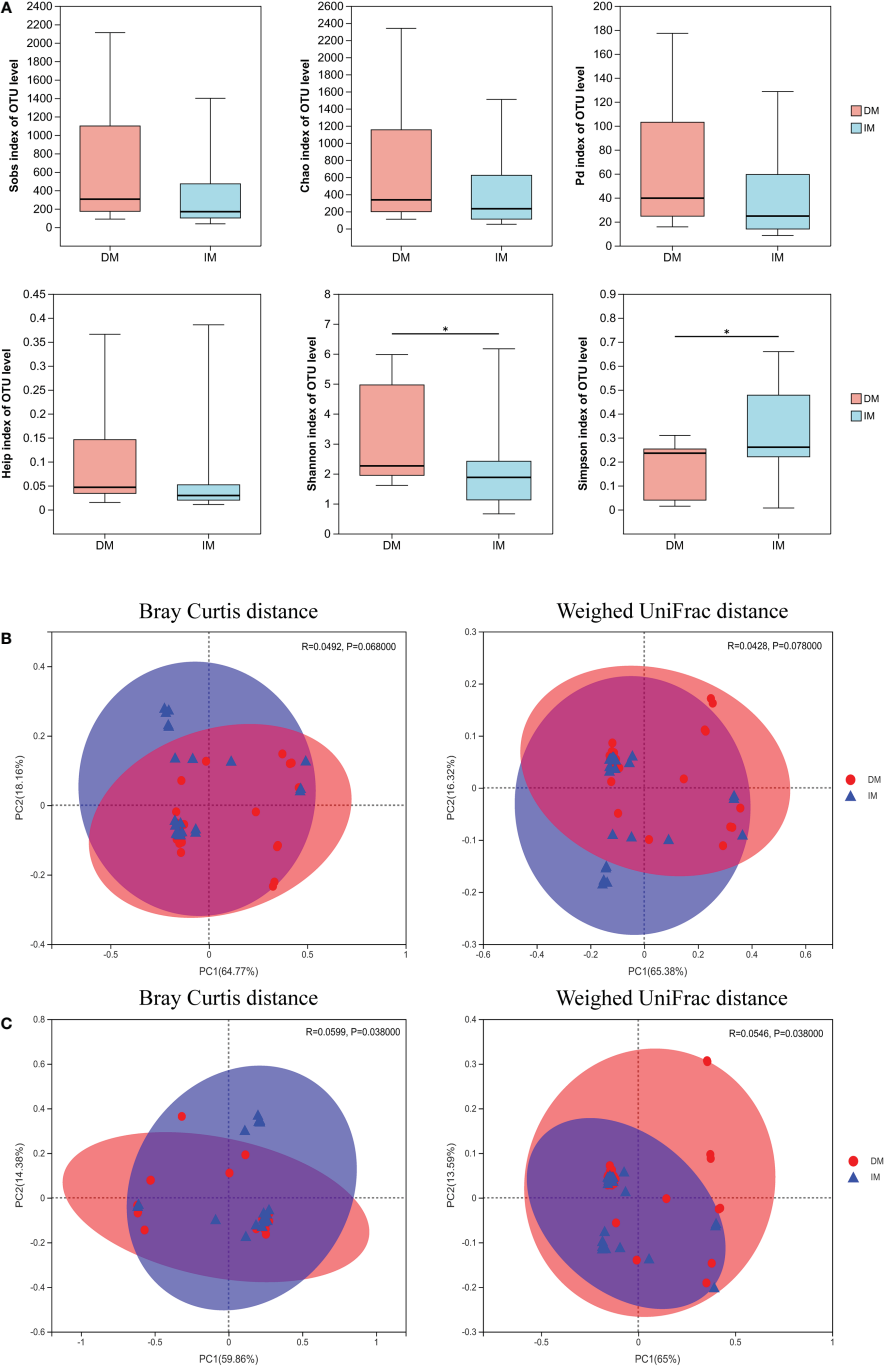
Tumor microbial alpha- and beta-diversity analysis based on influencing factors in both groups. **(A)** Microbial alpha diversity boxplot (observed OTUs, Sobs, Chao1, Pd, Heip, Shannon and Simpson indices) in SCRC patients. The DM (red) and IM (blue) groups (on the x-axis) and the different index types (on the y-axis). The PCOA plots of Bray Curtis and weighted UniFrac metric distances of beta diversity, **(B)** at the phylum level and **(C)** at the genus level. In the panel, each point represents a single DM (red circle) or IM (blue triangle), with ellipses (red ellipses= DM, blue ellipses = IM) representing the fitted mean and 95% confidence interval of each group, respectively. ^*^p < 0.05.

**Figure 4:**
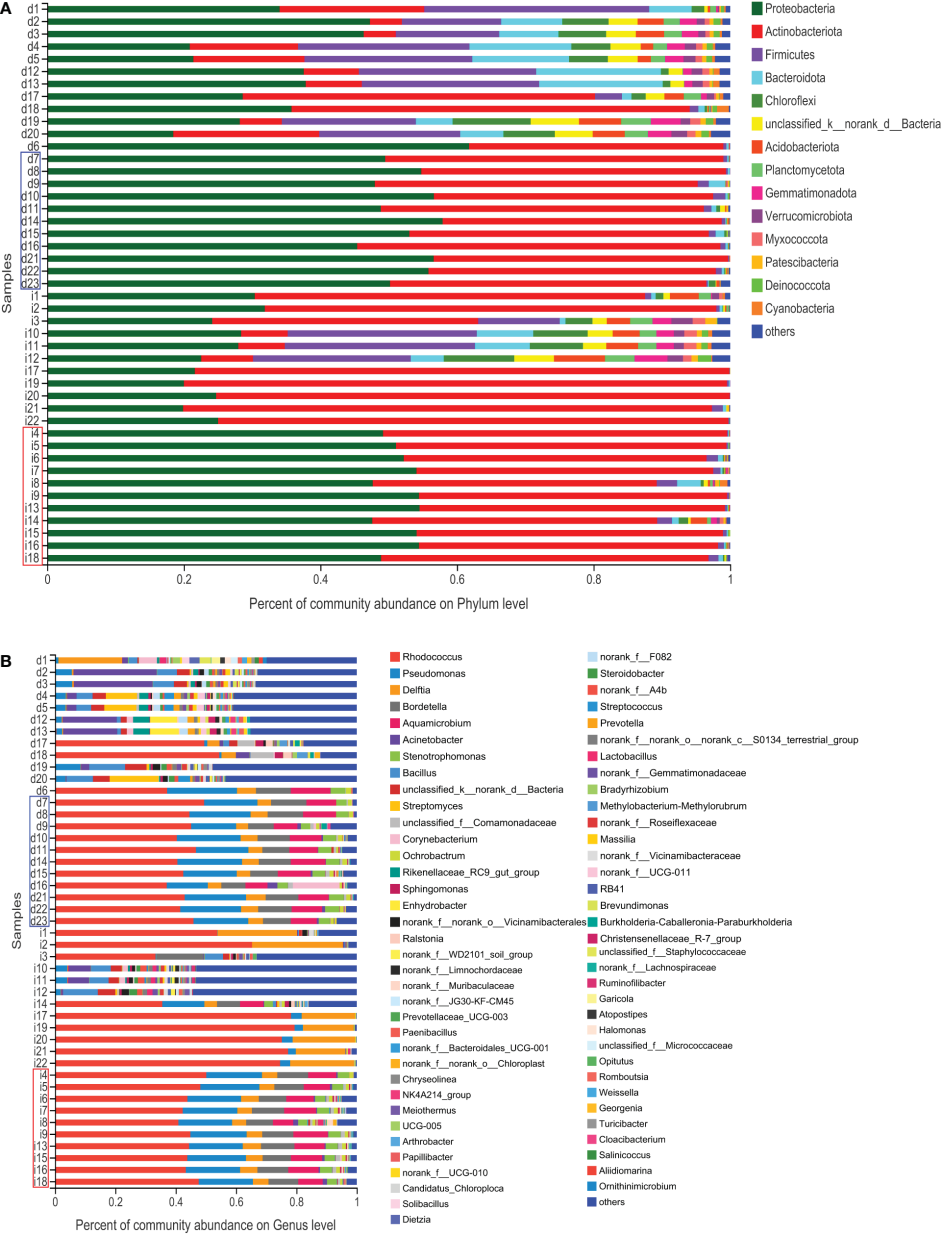
Histogram of the microbial community. The abundance of the community in each sample in the two groups. **(A)** At the phylum level. **(B)** At the genus level. The DM (red) and IM samples (blue) indicate similar abundances of microbial distribution.

A total of 2,421,631 high-quality sequences with an average length of 256 base pairs were generated. To determine whether the overall mean microbial community richness and diversity were different in negative/defective MLH1 (DM) versus positive/intact MLH1 (IM), we compared six measures of alpha diversity by sampling the following OTUs: Sobs and Chao1, which measure community richness; Shannon, Simpson, and phylogenetic diversity (PD) indices which estimate community diversity; and the Heip index, which describes the metric of community evenness (**Figure 3A**). The higher the value of Sobs and Chao1 index, the more the total number of species. There was a negative correlation between the Simpson index and community diversity, while a positive correlation between the Shannon index and community diversity. There were no significant differences in the Sobs, Chao1, Heip, and PD indices between DM and IM (p = 0.078, 0.071, 0.061, and 0.082, respectively); however, the Shannon and Simpson indices were significantly different (p = 0.049 and 0.025, respectively). The Shannon index indicated that the DM group showed a larger variation in α-diversity values than the IM controls, and the Simpson index showed the opposite trend; however, the two groups had more constant values in the other indices. To detect whether the overall enriched microbial communities were different between the two groups, we performed PCOA for each group. At the phylum level, analysis of SCRC cases stratified by immune phenotype revealed that there was no significant difference in bacterial relative OTUs between the two groups (p > 0.05, **Figure 3B**), as distinct clusters could not be noticed for both types of tumors in the comparison. At the genus level, the PCOA results illustrated a statistically significant difference for both Bray Curtis (p = 0.038) and weighted UniFrac (p = 0.038) measures (**Figure 3C**). Several sample nodes between the two groups overlapped in the PCOA distribution. On the basis of these observations, we plotted the proportion of microorganisms in each sample in the DM and IM groups to show the corresponding relationship and abundance of information more intuitively between both groups. Community investigation at the phylum (**Figure 4A**) and genus levels (**Figure 4B**) showed that some cases in both groups had similar sample nodes, which corresponded to their PCOA distribution. Collectively, clustering of community composition at different taxonomic levels was observed between the two groups of OTUs, indicating that the tumor microbiome of each group was similar in phylogeny.

### Tumor microbial communities are significantly different between DM and IM

**Figure 5:**
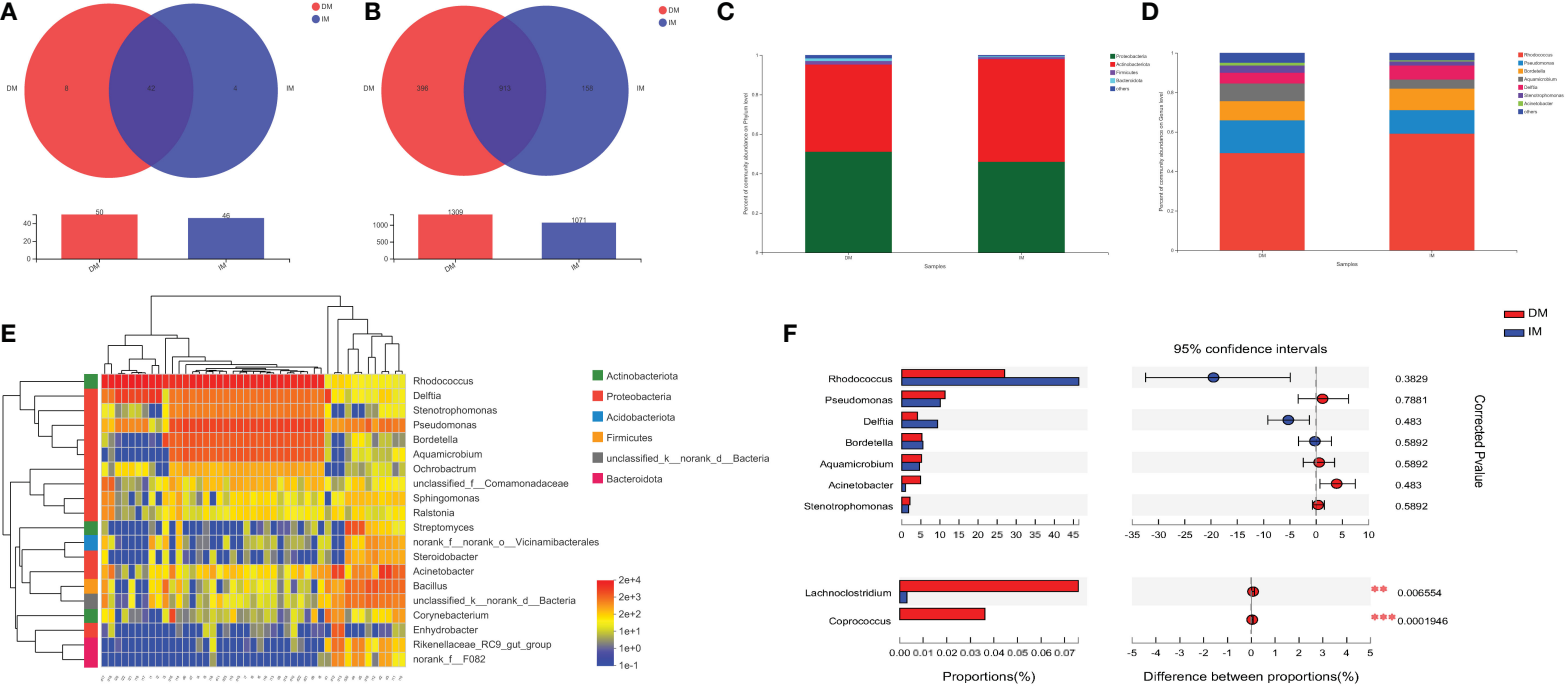
Tumor microbiome communities are significantly different between DM and IM. The Venn diagrams represent the shared and unique taxa among the different tissues by **(A)** the phyla cluster and **(B)** the genera cluster. **(C)** Dominant phyla of the two groups. **(D)** Dominant genera of the two groups. **(E)** Heatmap diagrams of microbial composition analysis identified top 20 genera with total abundance present across all SCRC samples. The relative abundance of species, with each represented by a different color. **(F)** At the genus level, the association between DM (red) and IM (blue) samples was detected by Wilcoxon signed-rank testing after correction for FDR. Selected taxa are shown with corrected p values (q). ^**^q < 0.01; ^***^q < 0.001.

After the taxa subsampling step, 54 phyla, 165 classes, 404 orders, 687 families, 1467 genera, 2842 species, and 6846 OTUs were identified in the 45 patients with SCRC, most of which were identified in the two sample types. With respect to the relationship between the intratissue bacterial diversity and protein phenotype of SCRC, we aimed to determine whether there were differences in tumor microbiome composition between the two groups. We first assessed the overall taxonomic profile of the tumor microbiota in all the patients, uncovering similar communities. **Figures 5A** and **5B** depict the results at the taxonomic level; the Venn diagram shows 42 phyla at the phylum level in both groups. Eight of the fifty phyla were unique to the DM group, compared with the IM group, whereas 384 of the 1327 genera were unique to the DM group.

Microbiomes with 1% relative abundance at different taxonomic levels were classified as “others”. At the phylum level, *Proteobacteria*, *Actinobacteria*, *Firmicutes*, and *Bacteroidetes* were the most common in the two groups during gut microbiome analysis, accounting for 98.51% and 99.42% of the total communities in DM and IM, respectively (**Figure 5C**). Seven members of the genera *Rhodococcus*, *Pseudomonas*, *Bordetella*, *Aquamicrobium*, *Delftia*, *Stenotrophomonas*, and *Acinetobacter*, were the most abundant in both groups, with proportions of 94.91% and 96.47% in DM and IM groups, respectively (**Figure 5D**). Subsequent analyses were adjusted for the top 20 genera with total abundance according to the hierarchical clustering of the microbiome belonging to seven phyla, including four dominant phyla (**Figure 5E**). Among them, *Rhodococcus* was the dominant genus from the phylum *Actinobacteria*, and the remaining dominant genera were from the phylum *Proteobacteria*. In addition, *Firmicutes* and *Bacteroidetes* were the predominant phyla with no corresponding dominant taxonomic composition at the genus level. Hence, all the above-mentioned species belonged to the predominant phyla (or genera) in the comparison of microbial composition in both groups and could comprise the specific microbiomes of both groups with different protein phenotypes.

To further compare whether there were microbial differences between the two groups, we performed the Wilcoxon rank-sum test. Because phyla-level clusters were relatively similar, no difference was reported between the two groups in this regard. In the analysis of the gut microbiome genome, we compared the relative abundance of individual taxa of the top seven dominant genera between the two groups and found three dominant genera (i.e., *Rhodococcus*, *Delftia*, and *Acinetobacter*) enriched by the Wilcoxon signed-rank test (p < 0.05, q > 0.05; **Table 3**). Notably, **Figure 5F** shows that these dominant genera were not significantly different after adjusting for FDR, and only the genera *Lachnoclostridium* and *Coprococcus* were significantly different in the comparison of all genera (q < 0.01 and q < 0.001, respectively). Correspondingly, the DM group showed markedly higher levels of *Lachnoclostridium* spp. and *Coprococcus* spp. as key species than the IM group, suggesting that these genera are associated with an increased MLH1 deficiency at the tumor site. These results suggest that the presence of these key species in the tumor may contribute to MLH1 protein deletion. Therefore, we propose a hypothesis that the lower abundance of bacteria in the DM group plays a more important role than that in the IM group, which may affect the development of SCRC.

**Table 3:** The relative abundances at the genus level and statistical significance between the DM and IM groups by FDR.

Genera	Proportion of Sequences (Means ± SD)			
	DM	IM	p-value	corrected p-value (q-value)
*Rhodococcus*	26.96 ± 22.22	46.49 ± 23.80	0.025^*^	0.383
*Pseudomonas*	11.36 ± 8.36	10.14 ± 8.12	0.563	0.788
*Bordetella*	5.34 ± 5.24	5.57 ± 5.38	0.334	0.589
*Aquamicrobium*	5.24 ± 5.22	4.66 ± 4.82	0.320	0.589
*Delftia*	4.10 ± 4.41	9.40 ± 8.98	0.044^*^	0.483
*Stenotrophomonas*	2.20 ± 2.12	1.83 ± 1.84	0.291	0.589
*Acinetobacter*	4.95 ± 8.49	1.05 ± 2.01	0.042^*^	0.483

FDR, false discovery rate. ^*^p < 0.05.

### Prediction of tumor microbiome phenotypes and various potential functions

**Figure 6:**
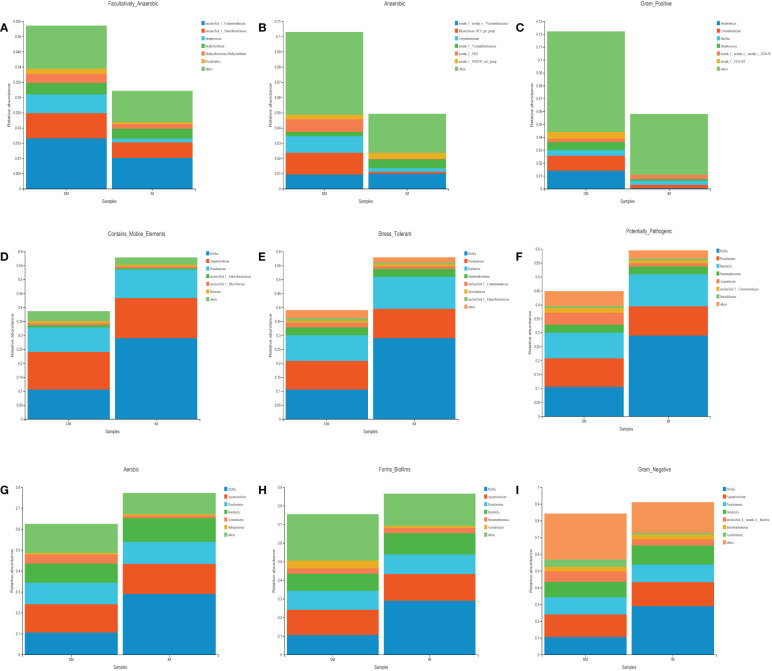
The analysis of intestinal species contribution to the phenotypic correlation in both groups. **(A)** Facultatively_Anaerobic. **(B)** Anaerobic. **(C)** Gram_Positive. **(D)** Contains_Mobile_Elements. **(E)** Stress_Tolerant. **(F)** Potentially_Pathogenic. **(G)** Aerobic. **(H)** Forms_Biofilms. **(I)** Gram_Negative.

**Figure 7:**
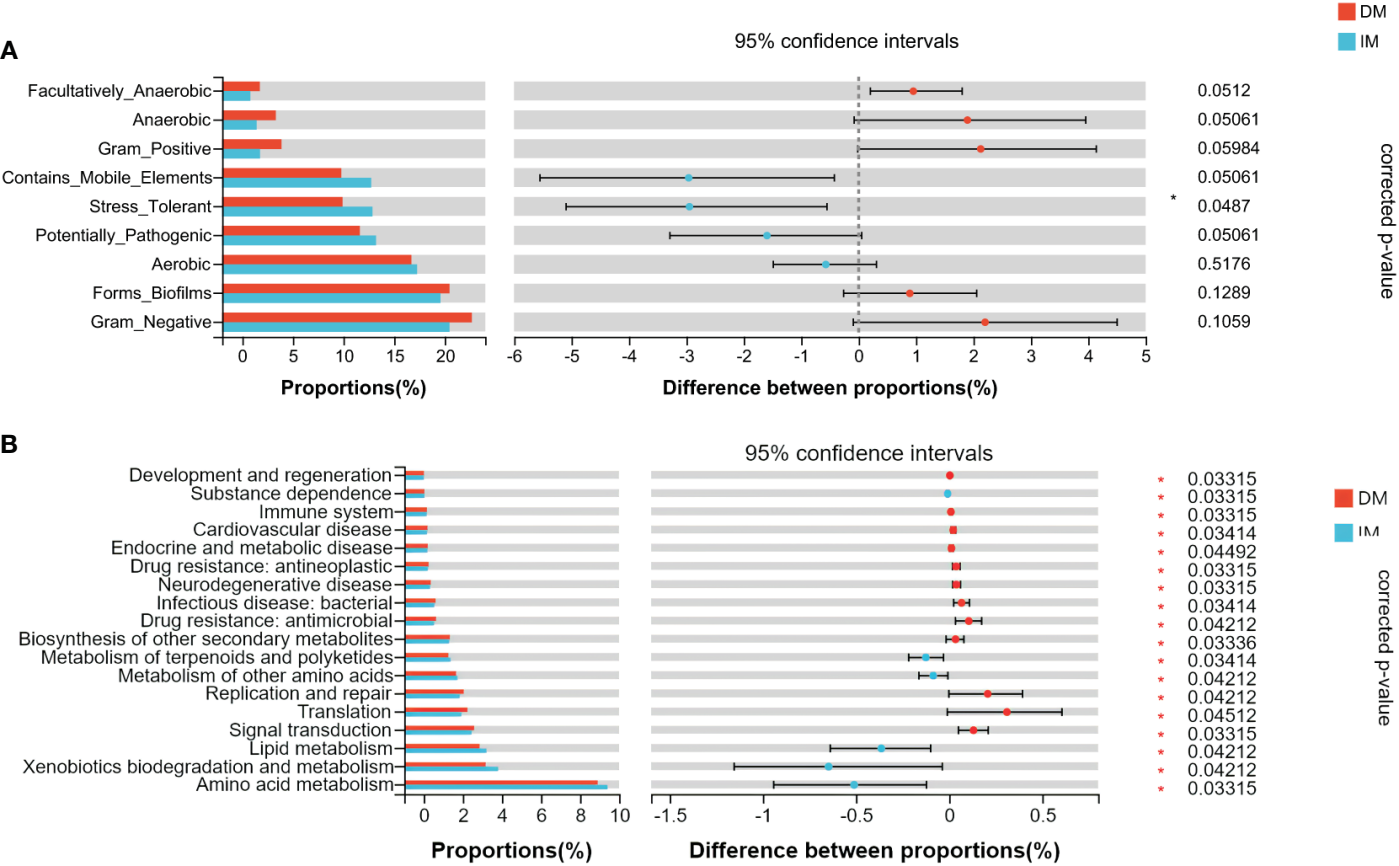
The difference of gut microbial phenotype and KEGG functional pathway between the two groups of SCRC patients. **(A)** The results of microbiome phenotype prediction by BugBase. **(B)** The significantly different KEGG pathways between the two groups by PICRUSt2. *q < 0.05.

The bacterial phenotype for each group was assessed using BugBase (**Figures 6A-I**). The statistical calculation of phenotypic abundance (**Figure 7A**) showed that after FDR correction, oxidative stress tolerance was significantly reduced in the DM group (q < 0.05). In addition, we used PICRUSt2 functional analysis to predict the metagenome and identified 18 level 2 KEGG pathways between the DM and IM groups (q < 0.05, **Figure 7B**). These functional genes were significantly different in both groups, belonging to five, level 1 KEGG pathways: metabolism, environmental information processing, genetic information processing, human disease, and biological system. When applied to the level 1 KEGG pathways, all SCRC samples had a coverage of more than 90% and q < 0.05. The DM group exhibited enrichment in pathways related to signal transduction, translation, replication and repair, biosynthesis of other secondary metabolites, antimicrobial drug resistance, bacterial infectious disease, neurodegenerative disease, antineoplastic drug resistance, endocrine and metabolic disease, cardiovascular disease, immune system, development, and regeneration. In contrast, the IM group showed enrichment in amino acid metabolism, xenobiotic biodegradation and metabolism, lipid metabolism, metabolism of other amino acids, terpenoids, and polyketides, and substance dependence. In the functional prediction analysis, it was found that the homologous genes of some prokaryotic microbiomes may be annotated to the metabolic pathway of eukaryotic organisms (39–41). In this case, we assume that the genes of these bacteria may be related to the two-component regulatory system, which will eventually reveal certain pathways that are not related to bacterial metabolism. The PICRUSt2 taxonomic and functional relationships indicate that the microbial community determines the differential enrichment of functional pathways between DM and IM, which may influence the MLH1 gene expression.

### Microbiome combination is significantly better than a single microbial factor in the diagnostic model

**Figure 8:**
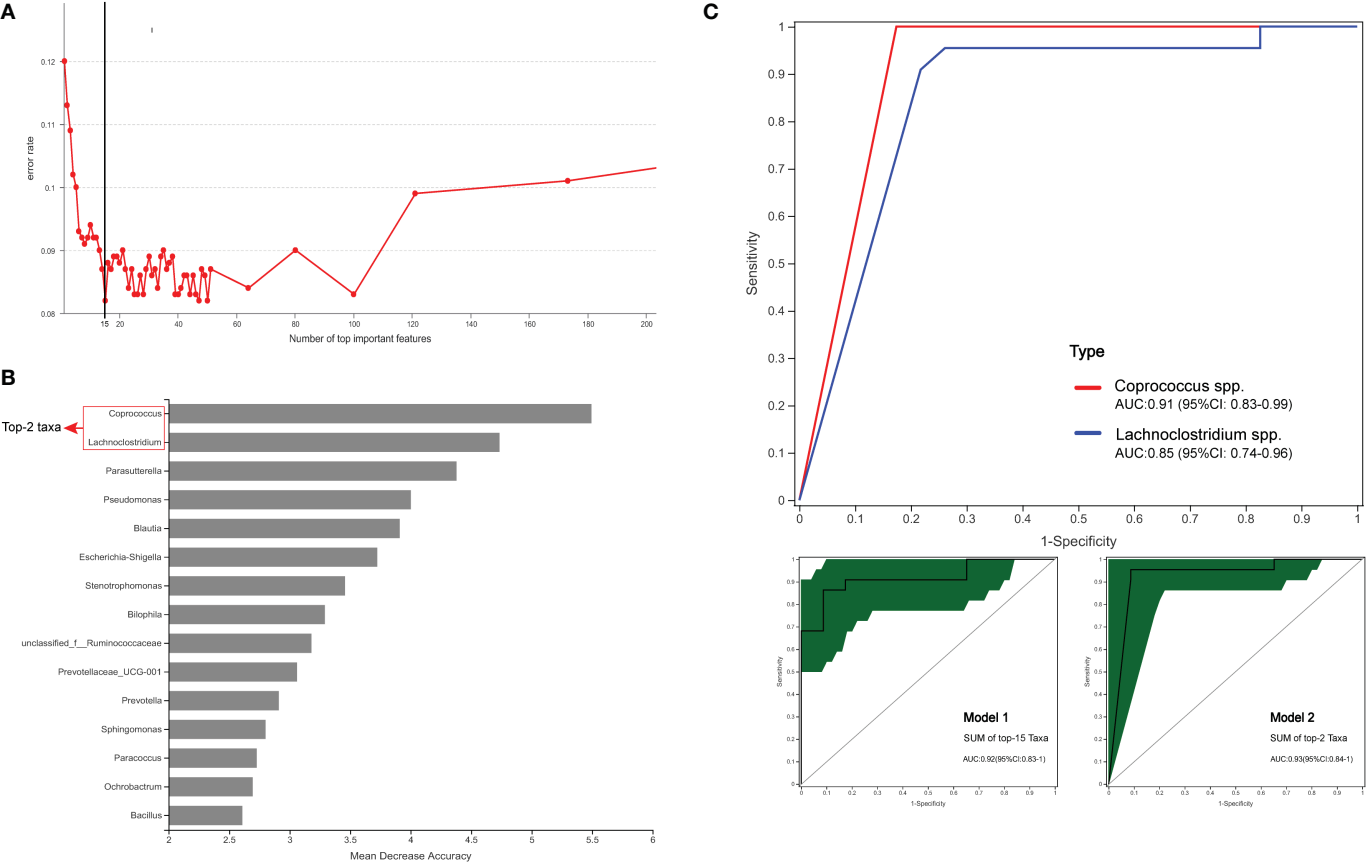
Comparison of bacterial (genus) candidate markers for the diagnostic prediction of DM SCRC. **(A)** The method of 10-fold cross-validation was used to determine the model with the lowest error rate of the ROC curve. **(B)** At the genus level, the relative abundance of the top15 hits, including *Lachnoclostridium* and *Coprococcus*. **(C)** ROC analysis of taxa relative abundance as predictive of DM status. The above figure shows the diagnostic performances of the bacteria (genus) *Lachnoclostridium* and *Coprococcus*. The figure below shows that Model 1 consists of the top 15 hits (left panel) and Model 2 consists of the top 2 hits (right panel). Different colors represent different groups of species.

This study explored whether key species can be used as biomarkers for disease diagnosis. The RF first classifies and predicts data information using the ROC method, which can then be used to evaluate the applicability and accuracy of the model classification. This study used the RF-ROC algorithm to determine the optimal model with maximum efficacy. As shown in **Figure 8A**, when the lowest error rate of the ROC curve was used, 15 species were selected for importance ranking. On sorting the top 15 model variations (**Figure 8B**), the genera *Coprococcus* (phylum *Firmicutes*) was ranked first, followed by remaining microbes in the microbiome, including *Firmicutes* phyla (including *Lachnoclostridium* spp., *Blautia* spp., *unclassified_f_Ruminococcaceae* spp., and *Bacillus* spp.), *Proteobacteria* phyla (including *Parasutterella* spp., *Pseudomonas* spp., *Escherichia-Shigella* spp., *Stenotrophomonas* spp., *Sphingomonas* spp., *Paracoccus* spp. and *Ochrobactrum* spp.), *Desulfobacterota* phyla (*Bilophila* spp.), and *Bacteroidetes* phyla (*Prevotellaceae UCG-001* spp. and *Prevotella* spp.).

Next, we attempted to assess the optimal biomarker candidates between single diagnostic prediction and multinomial combined model prediction (**Figure 8C**). We created Model 1, which illustrated the top 15 enriched genera, and had an AUC of 0.92 (95% CI: 0.83–1). We found that *Coprococcus* and *Lachnoclostridium*, as the top two genera, were significantly enriched in the DM group, with AUCs of 0.91 (95% CI: 0.83–0.99) and 0.85 (95% CI: 0.74–0.96), respectively. The results of the logistic regression model showed that Model 2 constituted the genera *Coprococcus* and *Lachnoclostridium*, with an AUC of 0.93 (95% CI: 0.84–1). These results demonstrated that Model 2 was the simplified optimal diagnostic model and was constructed by combining the top two biomarkers.

## Discussion

In this retrospective study, we sought to describe the gut microbiome of individuals with SCRC and compare the local microbiome of tumor tissues with different MLH1 expression levels. The comparison of SCRC patients with different protein expression levels revealed significant differences in the Shannon or Simpson indices, but not in the other indices. Importantly, this study showed two tumor microbial taxa as key genera, with *Lachnoclostridium* and *Coprococcus* being significantly enriched in DM patients. We compared the differences in the gut microbiome and predicted the gene function between the two groups. These bacteria were useful as biomarkers for DM SCRC, and a new panel of potential bacterial markers was designed, which may improve the clinical diagnosis of SCRC.

Recently, studies have shown that DMMR protein is present in approximately 15% of patients with CRC, and the predominant defective protein is MLH1 (42), which is similar to our study findings. Consequently, this study mainly focused on the microbiomes of tumors with MLH1 molecular characteristics assessed using 16S rRNA gene sequencing.

In line with the results of a previous study (43), the dominant gut microbiome composition in this study included *Actinobacteria*, *Proteobacteria*, *Firmicutes*, and *Bacteroidetes*. Some scholars have found that the degradation of dietary fiber by some species of the phylum Firmicutes could produce short-chain fatty acids (SCFAs), and butyric acid was one of the main components of SCFAs in animal assays (44, 45). Zhao et al. reported that the genus *Acinetobacter* (*Proteobacteria*) was positively correlated with butyrate for food microbes (46) and may be involved in reducing the risk of SCRC. Yang et al. performed a metagenomic analysis of the gut microbiome to assess the relationship between dietary habits and CRC, showing that grain-based diets reduced the risk of CRC and finding a significant reduction in the abundance of *Acinetobacter* as a candidate biomarker (47). Taken together, these findings demonstrate a complex system of associations between diet, the gut microbiome, and SCRC incidence, highlighting the role of the gut microbiome as a mediator.

Although gut microbial diversity is associated with the risk of SCRC, the key microbial roles that affected defective MLH1 in these tumors are not entirely clear. In this comparative study of the dominant genera, we found that the genera *Lachnoclostridium* and *Coprococcus* were positively correlated with defective MLH1 status. These microbes may play a role in inhibiting the MMR gene inactivation *via* the MLH1 expression pathway. Several studies have implicated that the genus-level microbiomes of *Lachnoclostridium* and *Coprococcus* are significantly different between patients with CRC and healthy cohorts (48, 49). Furthermore, Yuan et al. found a close correlation between *Lachnoclostridium* spp. and KRAS mutations using high-throughput sequencing of stool samples from patients with CRC and healthy controls (49). Previous studies have reported that some butyrate-producing bacteria of *Coprococcus* spp. (*Firmicutes*) could stimulate aberrant hyperproliferation of colonic epithelial cells with MSH2 or MLH1 deficiency and enhance β-catenin activity by increasing butyrate levels (50–52). Dronamraju et al. confirmed that butyrate has antitumor properties and can inhibit the proliferation of CRC cells with MLH1 gene deletion (53). As stated above, specific bacteria in the gut microbial community may induce MLH1 mutation to facilitate the occurrence of SCRC.

We speculate that the characteristics of tumor-associated microorganisms may trigger MLH1 protein deficiency in patients with SCRC and further promote the development of MSI. An understanding of relevant functional genes is required to explain this phenomenon and predict the role of these bacteria in SCRC for different MLH1 phenotypes. With the BugBase phenotypic prediction, we observed that, in patients with SCRC, oxidative stress tolerance was negatively correlated with DM and positively associated with IM. The oxidative stress response in the human body results in the enrichment of reactive oxygen species and destroys the biological molecular structure, which may eventually lead to cancer (54, 55). Qiao et al. indicated that the imbalance of flora in the diet might be caused by oxidative stress, and the changes in lactic acid bacteria, *Lactobacillus*, *E. coli*, and *Enterococci* in the intestine played a significant role (56). In contrast, our results indicate that, at the genus level, the microorganism most likely to be associated with oxidative stress is *Delftia*. In terms of functional prediction, the study showed that functional genes with the highest abundance in units with significant differences were those involved in amino acid metabolism. Previously, few researchers have reported that among the most enriched pathways, the pathway related to amino acid metabolism is closely associated with CRC (57). Based on the prediction analysis of the KEGG molecular function database, we retrieved partially functional genes in *Pseudomonas* spp. that were strongly linked to multiple level 2 KEGG pathways (such as replication and repair, translation, and signal transduction); this finding is supported by the results of Shen et al. (58). Regarding the mechanism of MLH1 protein deletion in patients with SCRC, the current relatively established theory suggests that functional pathways such as protein biosynthesis, KEGG metabolism pathways, the ammonia cycle, and galactose metabolism are the factors leading to MLH1 promoter hypermethylation (59, 60). Hence, multiple functional predictions elucidated the relationship between microbiome sequencing abundance and tumor-related MLH1 protein phenotype and may be beneficial for further determination of metabolic biomarkers derived from patients with DM.

Testing analysis of fecal samples from 1012 Asian participants showed that the abundance of *Lachnoclostridium* spp. was increased in intestinal adenoma and was a novel non-invasive genetic biomarker for the diagnosis and prediction of intestinal tumors (61). As a validation, Liang et al. identified that the combined predictive analysis of *Lachnoclostridium* and two bacterial markers (*Fusobacterium nucleatum* and *Clostridium hathewayi*) could improve the sensitivity and specificity of recurrent adenoma diagnosis (62). However, to date, it is not clear whether microbial communities affect clinical practice in CRC tissues with an MLH1 protein phenotype. We found that patients who had SCRC with candidate gene biomarkers had a greater probability and higher biological susceptibility of developing MSI-H/DMMR. To this end, we further identified a new dataset of bacterial markers for patients with DM; the AUC value of the most highly predictive model was 0.92, obtained using RF-ROC analysis. By stepwise testing of the species model at the genus level, compared with the microbiome alone, the combination of *Lachnoclostridium* and *Coprococcus* demonstrated an increased AUC-ROC, indicating the impact of the bacterial combination on improving other candidate biomarkers for diagnostic prediction of DM SCRC. Surprisingly, we developed a simplified optimal model in the present study that required only two biomarkers to maintain an AUC of 0.93, demonstrating that these genera have a high discriminative power to evaluate the risk of patients developing DM. Additionally, we believe that if *Lachnoclostridium* spp. and *Coprococcus* spp. are the major variants, they can be used for clinical diagnosis prediction in patients with DM and have a high reference value. The results of such random forest analysis need to be interpreted with caution when small sample sizes are constructed for model predictive analysis.

Unfortunately, this retrospective study had some limitations. First, the low incidence of DM status in intestinal tumor tissue limited the number of patients with DM-SCRC who were enrolled; thus, the findings of this study need to be validated. Second, more clinical samples need to be included in future studies because differences in regions, eating habits, races, and ethnicities could potentially limit the generalizability of the study. Third, we analysed the microbiome within tumor tissues only in patients with SCRC without examining paired adjacent normal tissues or synchronously examining other microbiome samples (such as stool). Fourth, the causality between disease-related protein phenotypes and microbial diversity could not be well established. We continuously collected tissue samples from patients with DM, obtaining sequence data with as large a sample as possible to validate candidate bacterial markers in future studies of microbiomes based on disease diagnostic models.

## Conclusion

We found that MLH1 gene silencing is the predominant role of DMMR in the tumor tissues of patients with SCRC. The relative abundance of the gut microbiome differs significantly in the tumor microenvironment between DM and IM patients with SCRC. We also evaluated the bacterial phenotype and flora function relative to tumor microbiota in both groups. More importantly, we also designed a simplified optimal model (i.e., the combination of *Lachnoclostridium* spp. and *Coprococcus* spp.), which is a new non-invasive index to predict the clinical adjuvant efficacy and prognosis of SCRC by predicting the independent risk factors for MLH1 mutation phenotype and may provide a novel direction for future prognostic diagnosis and immunotherapy in patients with SCRC.

## Data availability statement

The datasets presented in this study can be found in online repositories. The names of the repository/repositories and accession number(s) can be found in the article/**Supplementary Material**.

## Ethics statement

The studies involving human participants were reviewed and approved by the Ethics Committee of Harbin First Hospital (Approval No. Q2021-064). The patients/participants provided their written informed consent to participate in this study.

## Author contributions

All authors made substantive intellectual contributions to the present study and approved the final manuscript. AZ designed the study and wrote the draft manuscript. YL, AZ, ZL and YHe coordinated patient recruitment and sample collection. AZ and LB conducted bioinformatic analysis. YW and YZ conducted 16S rRNA gene sequencing. PJ and YHu contributed to the data interpretation and the revision of the manuscript. All authors participated in the coordination of the study and approved the final manuscript. All authors contributed to the article and approved the submitted version.

## Footnotes

1
https://ccb.jhu.edu/software/FLASH/index.shtml

2
http://www.drive5.com/usearch/

3
http://rdp.cme.msu.edu/

4
https://www.arb-silva.de/

5
https://bugbase.cs.umn.edu/index.html

6
https://github.com/picrust/picrust2/
