# Supplementary figures and images for: Diagnosis and functional prediction of microbial markers in tumor tissues of sporadic colorectal cancer patients associated with the MLH1 protein phenotype
Source: Front Oncol. 2023 Jan 23;12:1116780. doi: 10.3389/fonc.2022.1116780 (PMC9899897; doi:10.3389/fonc.2022.1116780)

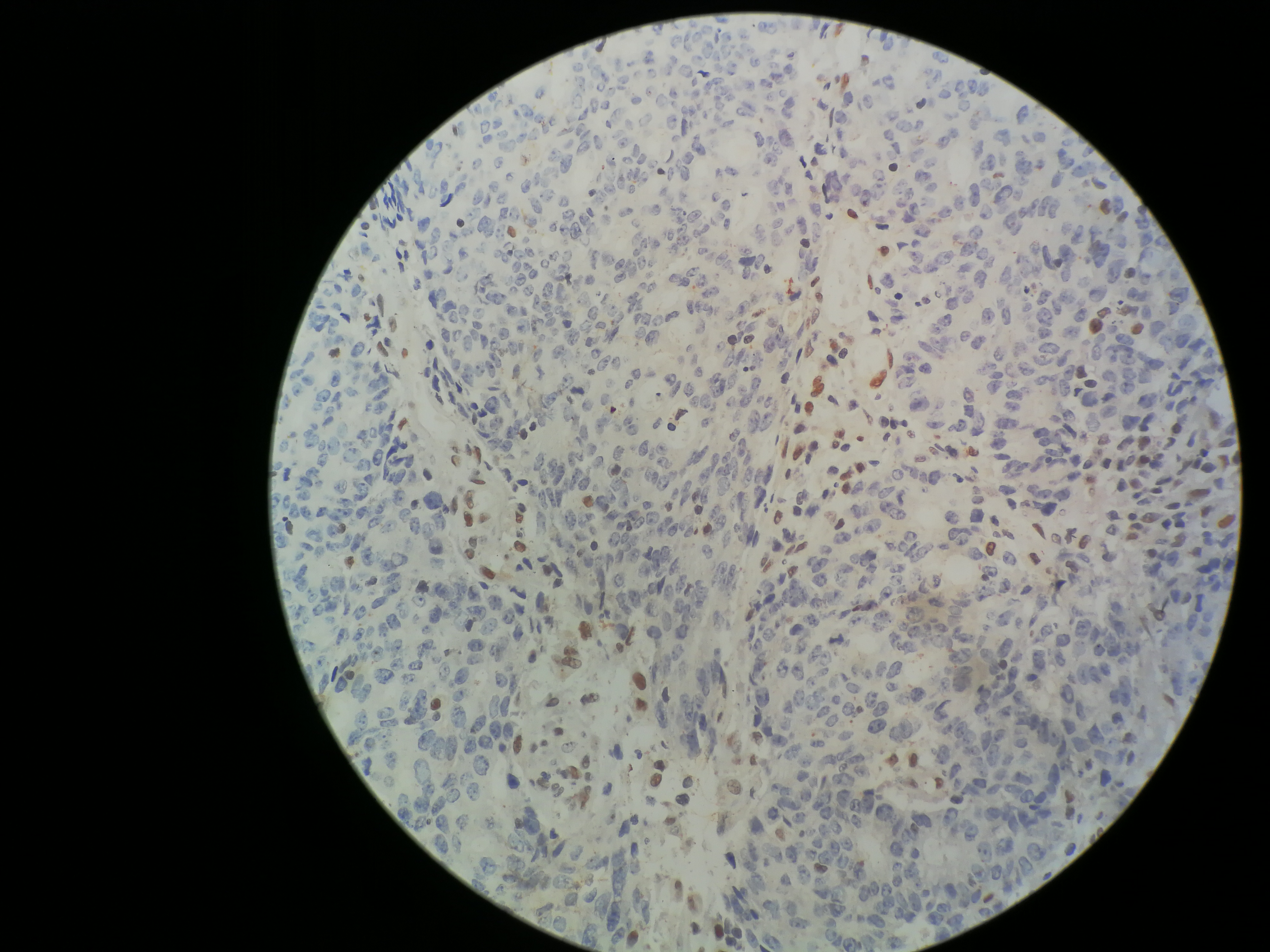

Supplement: Supplementary file 2 [file DataSheet_2.zip › Original photo/DM MLH1.jpg]

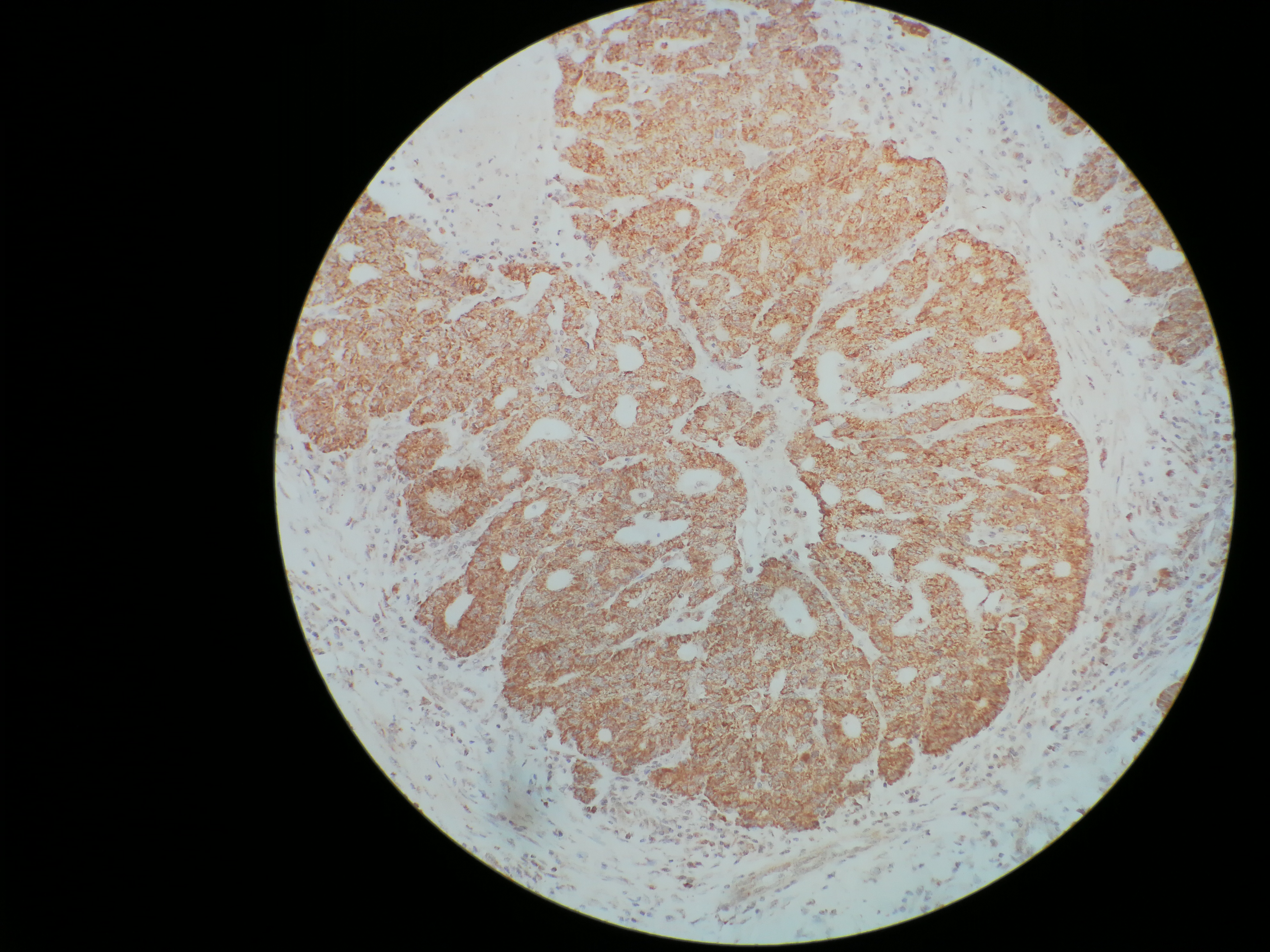

Supplement: Supplementary file 2 [file DataSheet_2.zip › Original photo/DM BRAF.jpg]

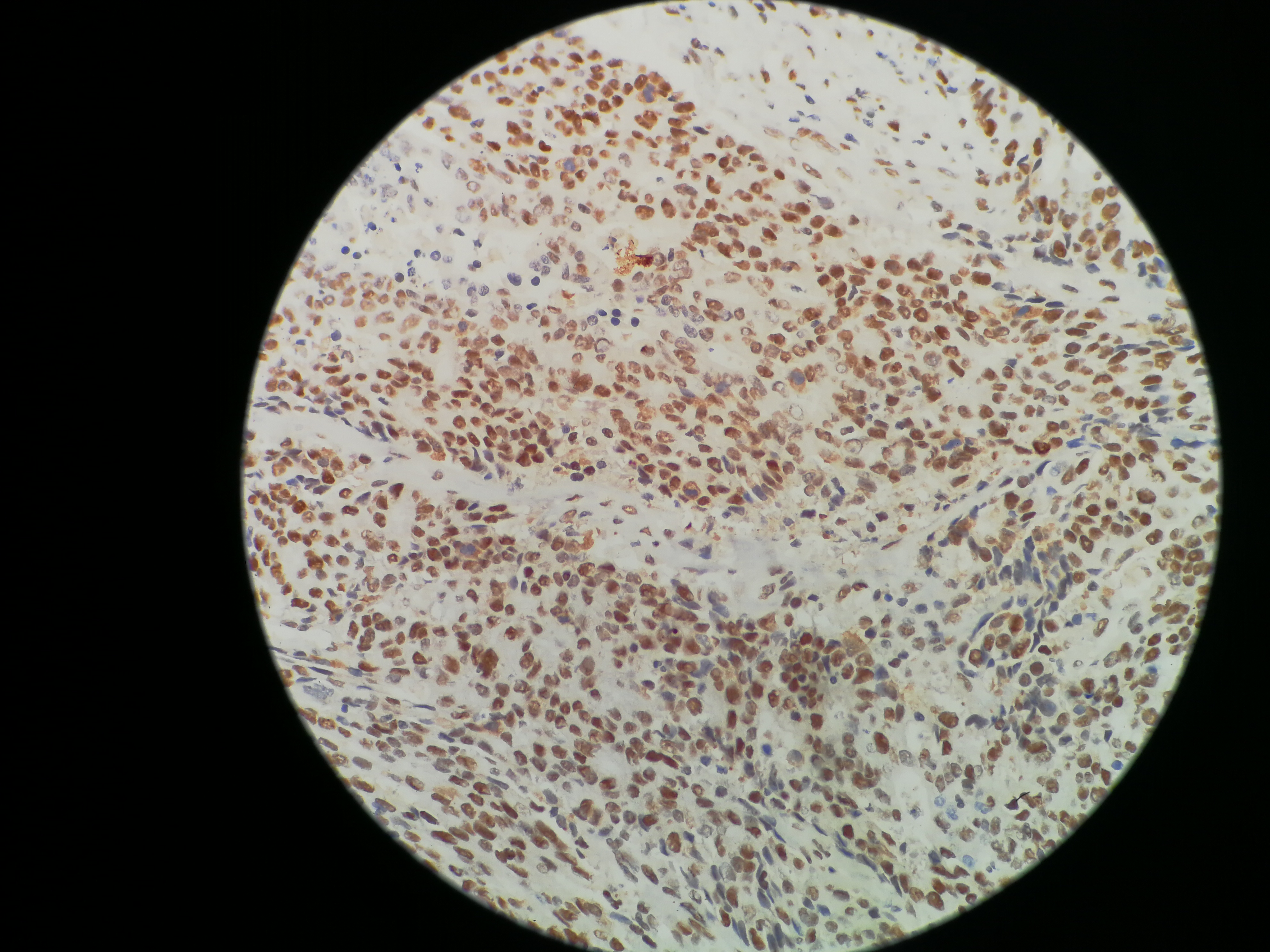

Supplement: Supplementary file 2 [file DataSheet_2.zip › Original photo/DM MSH2.jpg]

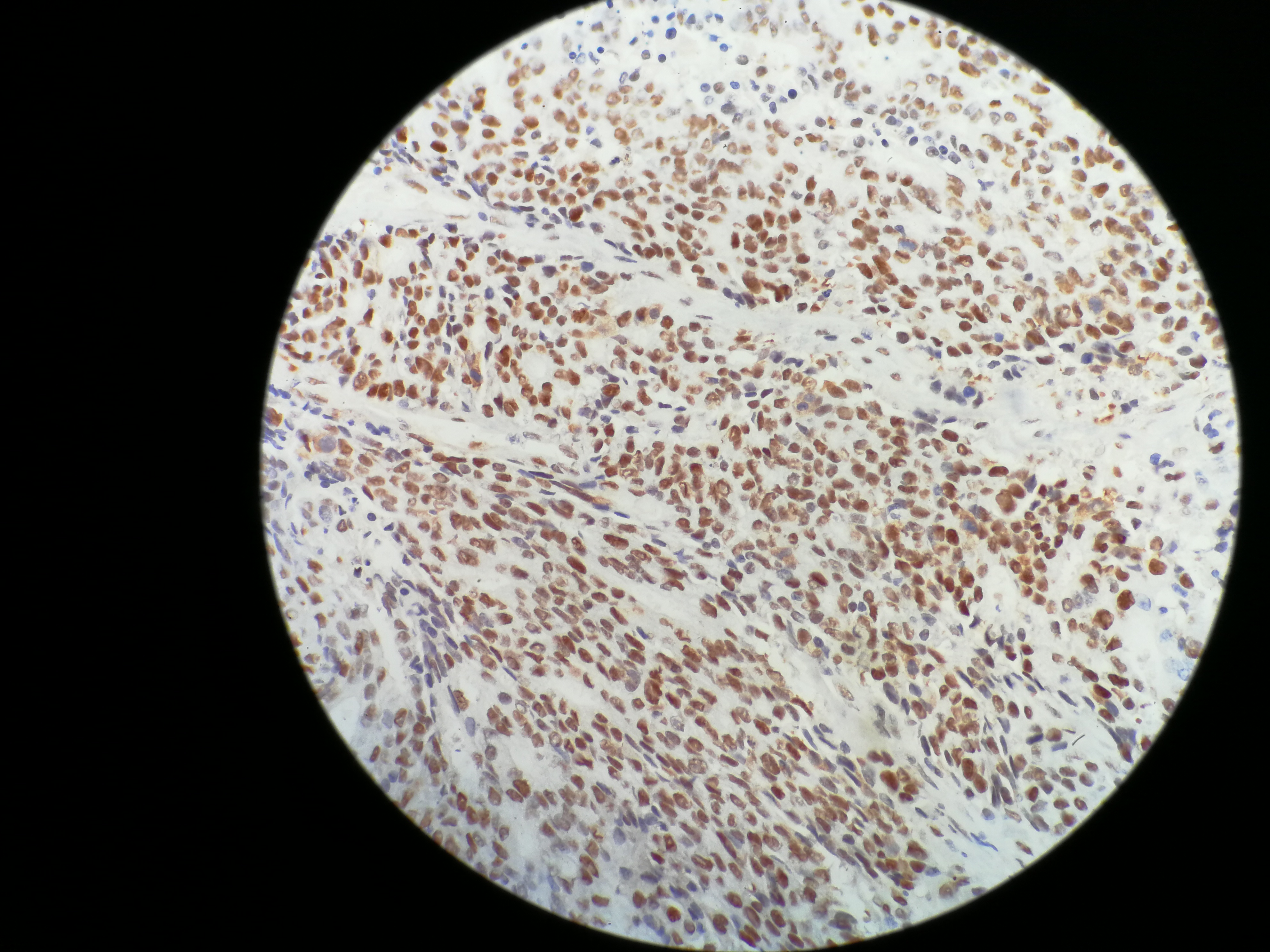

Supplement: Supplementary file 2 [file DataSheet_2.zip › Original photo/DM MSH6.jpg]

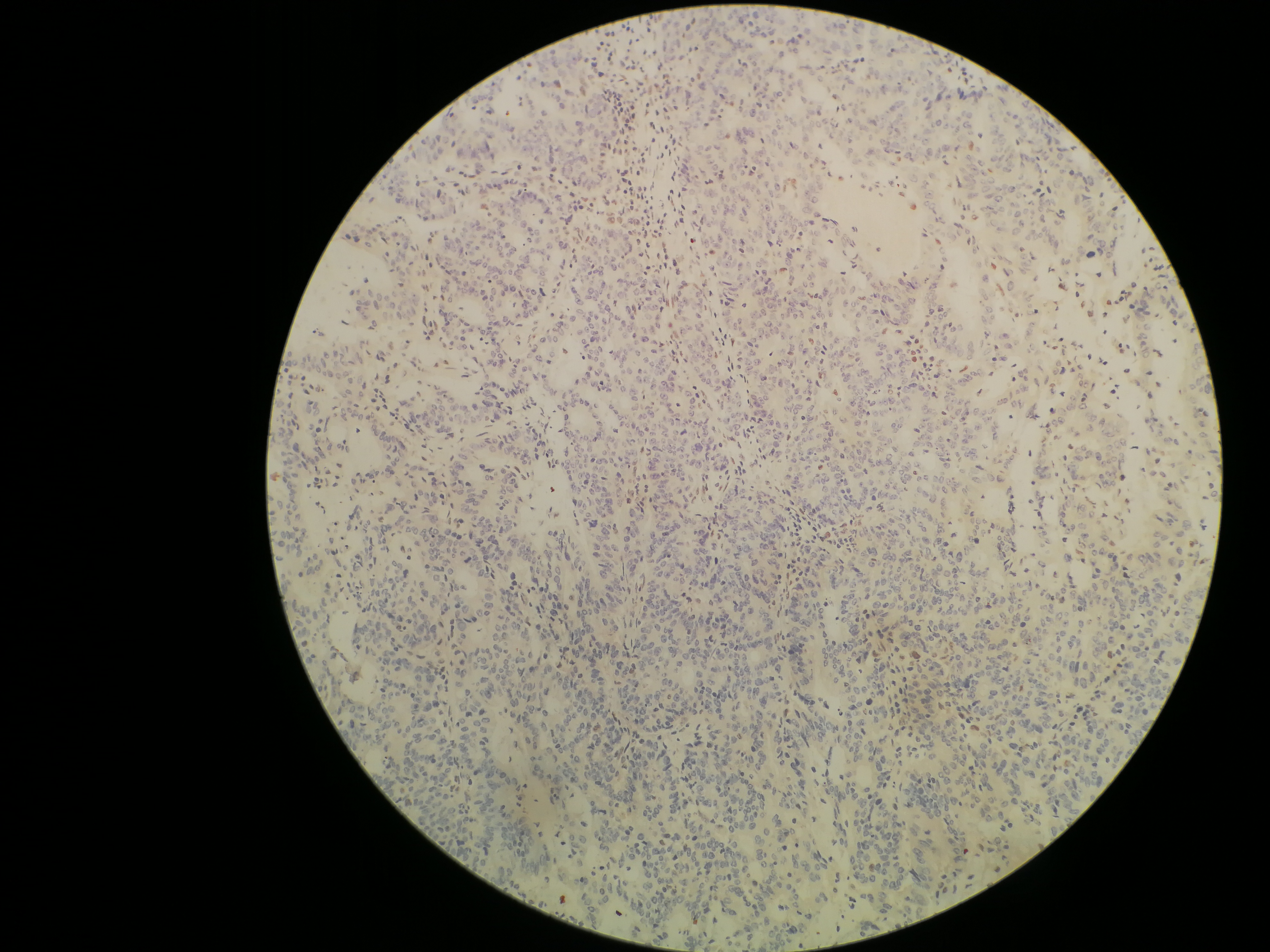

Supplement: Supplementary file 2 [file DataSheet_2.zip › Original photo/DM PMS2.jpg]

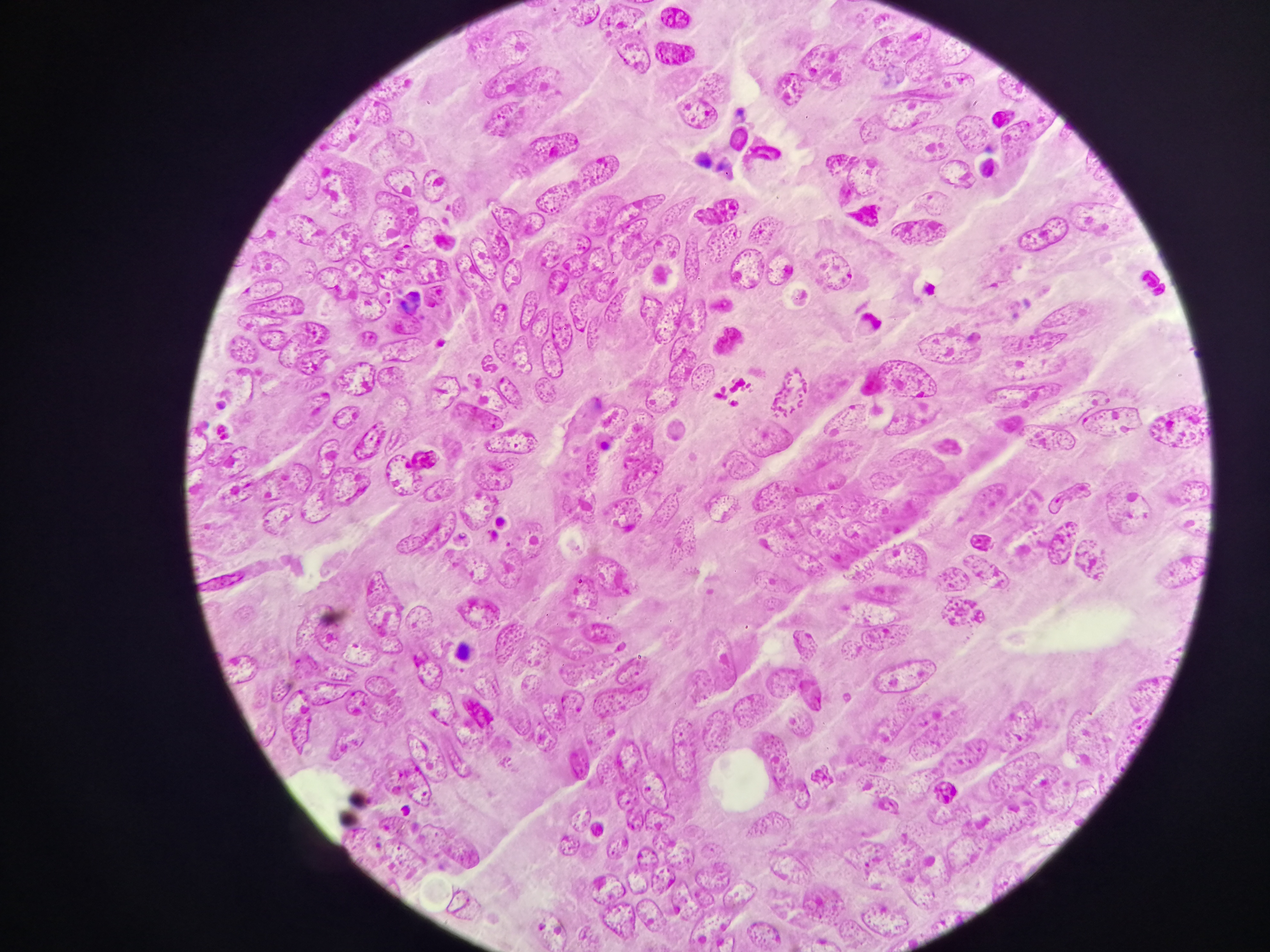

Supplement: Supplementary file 2 [file DataSheet_2.zip › Original photo/Gram staining DM.jpg]

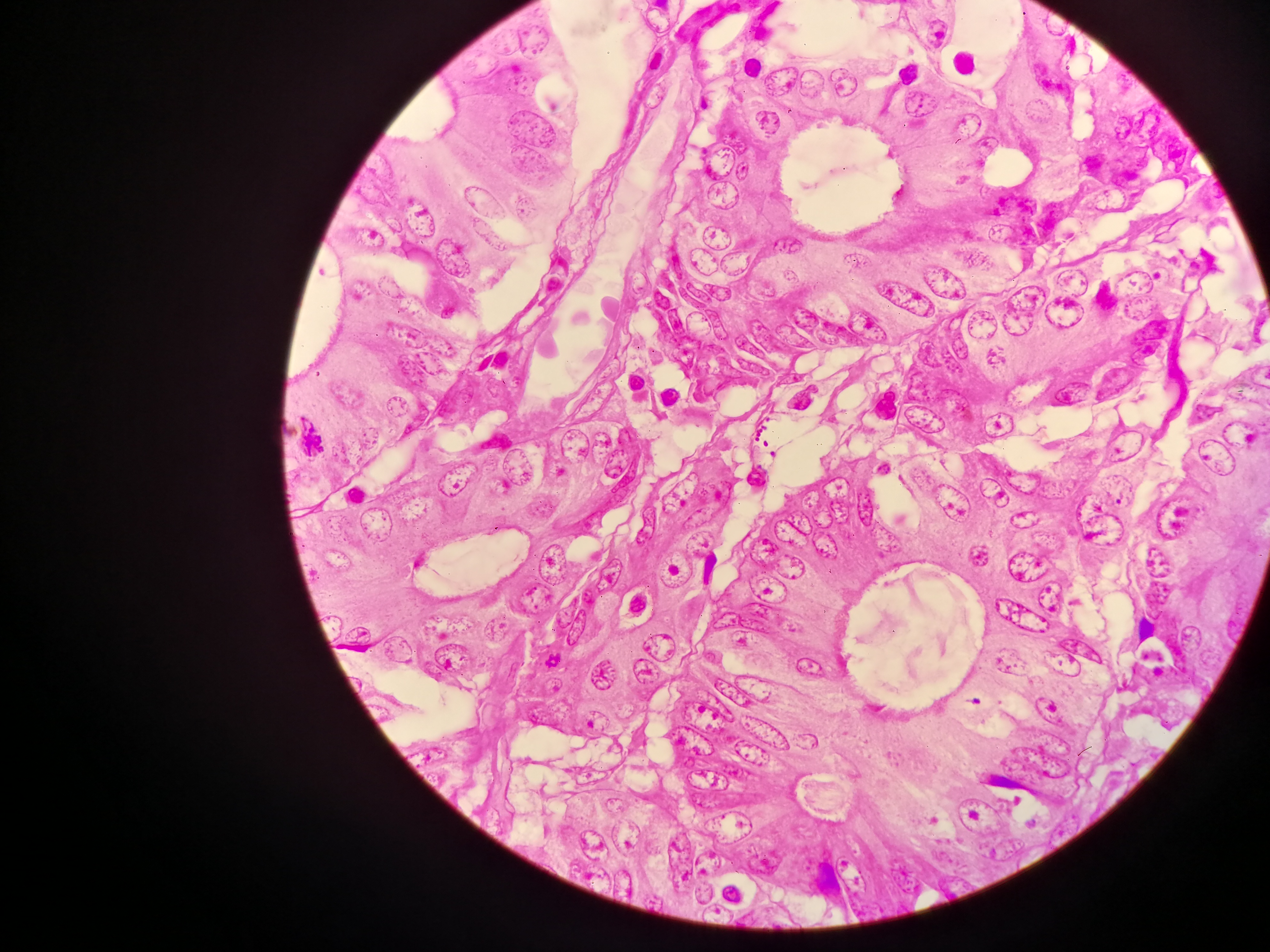

Supplement: Supplementary file 2 [file DataSheet_2.zip › Original photo/Gram staining IM.jpg]

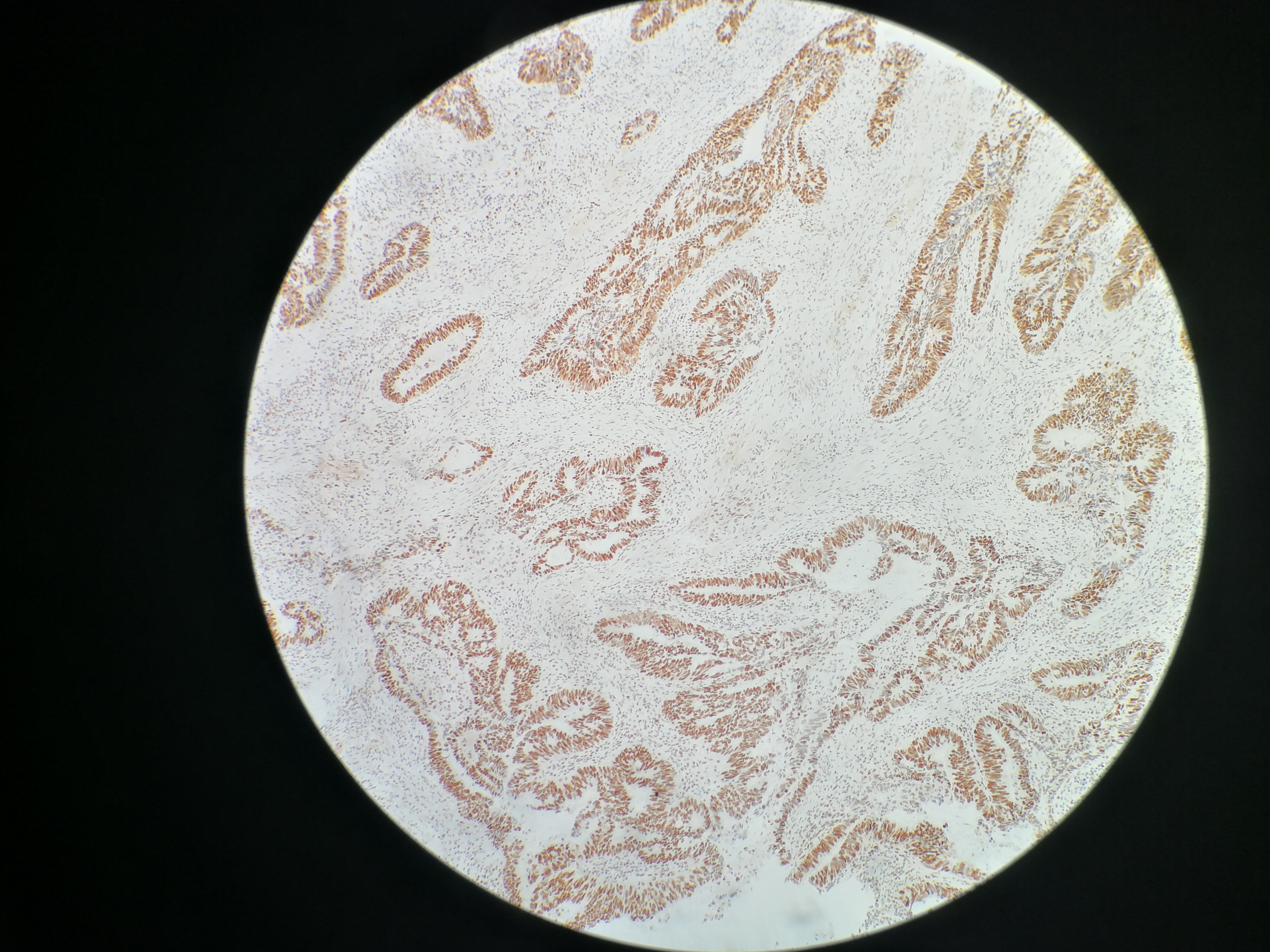

Supplement: Supplementary file 2 [file DataSheet_2.zip › Original photo/IM MLH1.jpg]

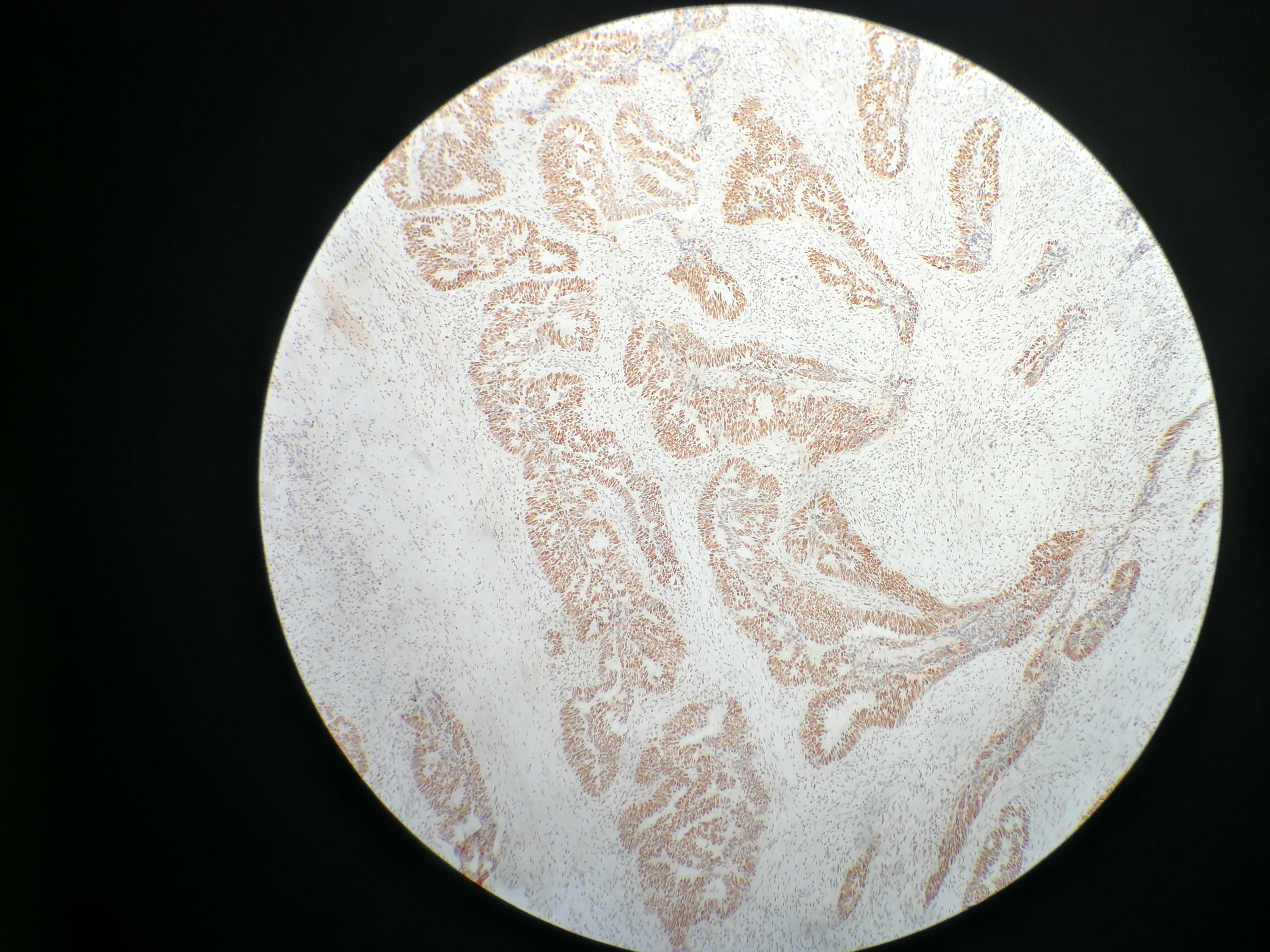

Supplement: Supplementary file 2 [file DataSheet_2.zip › Original photo/IM MSH2.jpg]

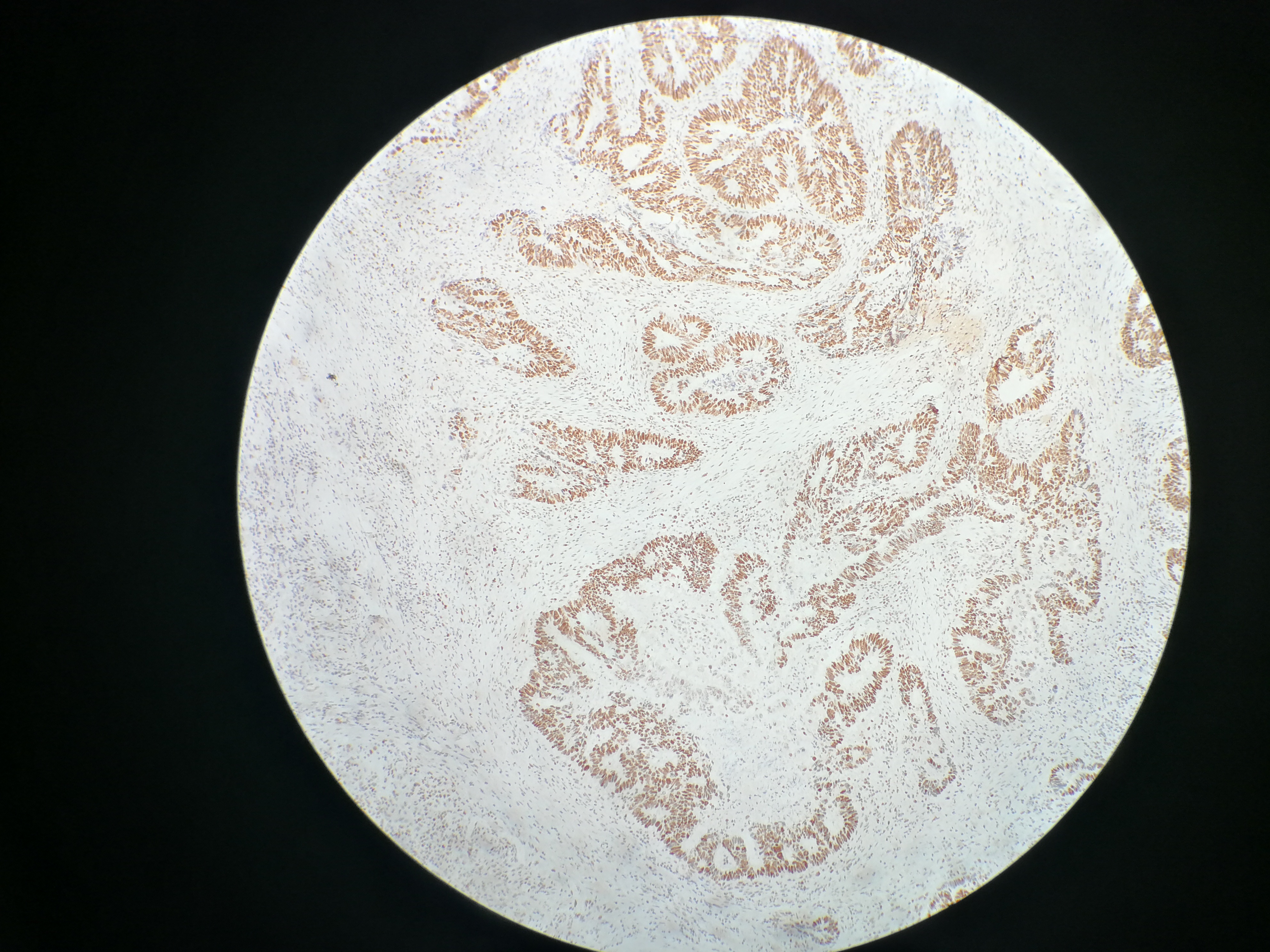

Supplement: Supplementary file 2 [file DataSheet_2.zip › Original photo/IM MSH6.jpg]

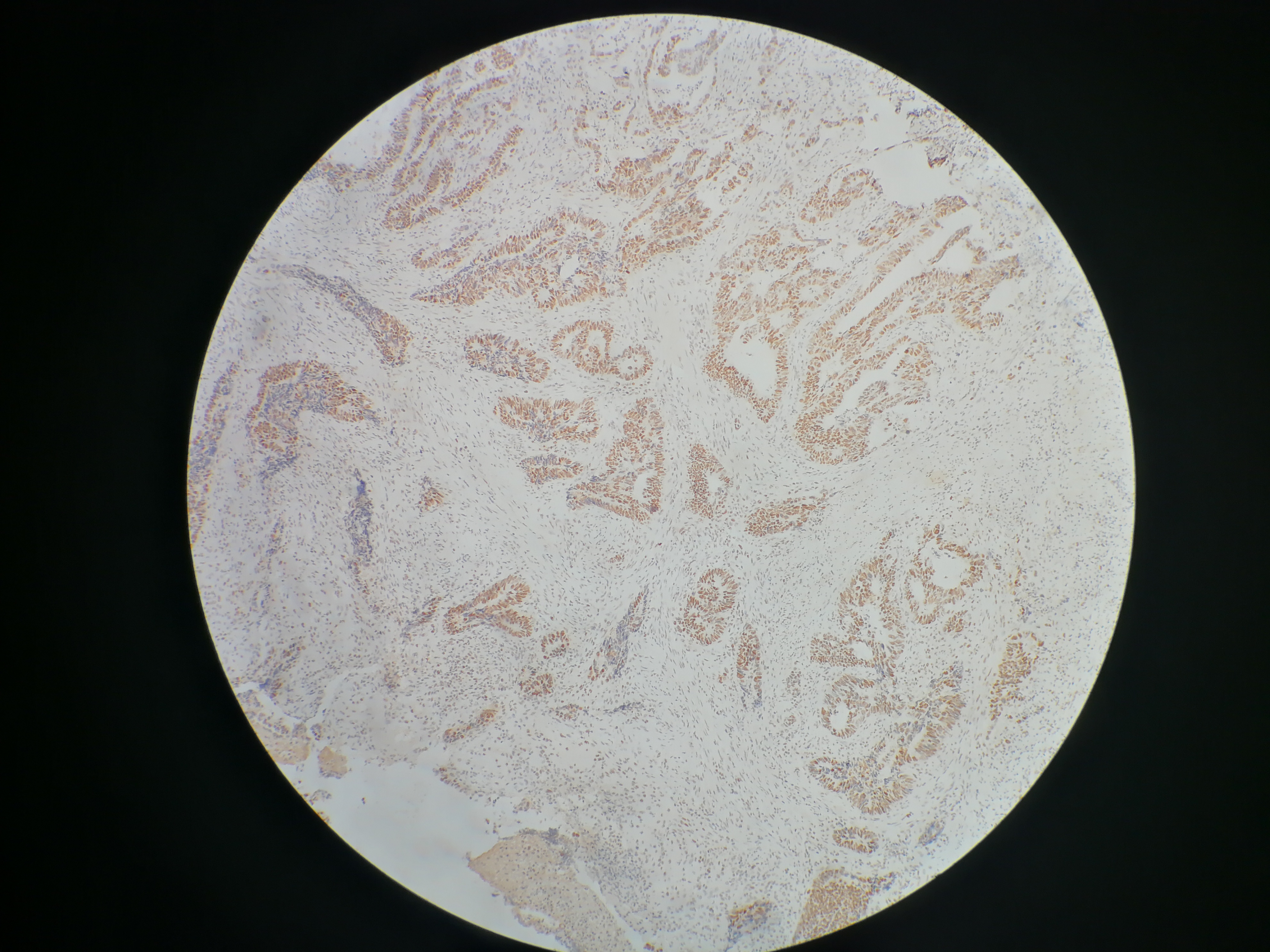

Supplement: Supplementary file 2 [file DataSheet_2.zip › Original photo/IM PMS2.jpg]
